# Computational Flux Balance Analysis Predicts that Stimulation of Energy Metabolism in Astrocytes and their Metabolic Interactions with Neurons Depend on Uptake of K^+^ Rather than Glutamate

**DOI:** 10.1007/s11064-016-2048-0

**Published:** 2016-09-14

**Authors:** Mauro DiNuzzo, Federico Giove, Bruno Maraviglia, Silvia Mangia

**Affiliations:** 1 Center for Basic and Translational Neuroscience, Faculty of Health and Medical Sciences, University of Copenhagen, Blegdamsvej 3B, 24.2.40, 2200 Copenhagen N, Denmark; 2 Museo Storico della Fisica e Centro Studi e Ricerche “Enrico Fermi”, Rome, Italy; 3 Fondazione Santa Lucia IRCCS, Rome, Italy; 4 Center for Magnetic Resonance Research, Department of Radiology, Univeristy of Minnesota, Minneapolis, MN USA

**Keywords:** Brain energy metabolism, Potassium, Neuron-astrocyte interactions, Constraint programming, Flux balance analysis

## Abstract

Brain activity involves essential functional and metabolic interactions between neurons and astrocytes. The importance of astrocytic functions to neuronal signaling is supported by many experiments reporting high rates of energy consumption and oxidative metabolism in these glial cells. In the brain, almost all energy is consumed by the Na^+^/K^+^ ATPase, which hydrolyzes 1 ATP to move 3 Na^+^ outside and 2 K^+^ inside the cells. Astrocytes are commonly thought to be primarily involved in transmitter glutamate cycling, a mechanism that however only accounts for few % of brain energy utilization. In order to examine the participation of astrocytic energy metabolism in brain ion homeostasis, here we attempted to devise a simple stoichiometric relation linking glutamatergic neurotransmission to Na^+^ and K^+^ ionic currents. To this end, we took into account ion pumps and voltage/ligand-gated channels using the stoichiometry derived from available energy budget for neocortical signaling and incorporated this stoichiometric relation into a computational metabolic model of neuron-astrocyte interactions. We aimed at reproducing the experimental observations about rates of metabolic pathways obtained by ^13^C-NMR spectroscopy in rodent brain. When simulated data matched experiments as well as biophysical calculations, the stoichiometry for voltage/ligand-gated Na^+^ and K^+^ fluxes generated by neuronal activity was close to a 1:1 relationship, and specifically 63/58 Na^+^/K^+^ ions per glutamate released. We found that astrocytes are stimulated by the extracellular K^+^ exiting neurons in excess of the 3/2 Na^+^/K^+^ ratio underlying Na^+^/K^+^ ATPase-catalyzed reaction. Analysis of correlations between neuronal and astrocytic processes indicated that astrocytic K^+^ uptake, but not astrocytic Na^+^-coupled glutamate uptake, is instrumental for the establishment of neuron-astrocytic metabolic partnership. Our results emphasize the importance of K^+^ in stimulating the activation of astrocytes, which is relevant to the understanding of brain activity and energy metabolism at the cellular level.

**Electronic supplementary material:**

The online version of this article (doi:10.1007/s11064-016-2048-0) contains supplementary material, which is available to authorized users.

## Introduction

One major focus of neuroenergetics research is directed towards the interactions between neuronal and glial cells, the major cellular constituents of the central nervous system. Signaling by neurons and protoplasmic astrocytes indeed controls information processing in the cortical grey matter of the brain. It is now recognized that neurophysiological mechanisms operate under a number of constraints, including cell-specific reaction/transport processes and their dependence on substrate and energy availability [1, 2]. In particular, these constraints involve delicate balances between substrate supply and demand within specialized cellular metabolic networks counting dozens of coupled biochemical reactions. Although the experimental methods face this complexity with progressively improved technical strategies, computational models remain useful tools in providing interpretative insights to experimental data [3]. In particular, standard as well as probabilistic stoichiometric models have been applied to the study of compartmentalized brain energy metabolism [4–14]. These works have provided valuable insights into specific aspects of neuron-astrocyte interactions, however none have incorporated explicit pathways involved in ion homeostasis (i.e. pumps and channels) related to neurotransmission (see discussion in [6]). In fact, ionic species were present in some stoichiometric models (e.g., [12]) but they were related neither directly nor indirectly with neuronal glutamatergic signaling activity. In the present work we overcame this limitation by taking into account ionic movements across brain cells and the associated energy demand.

The primary aim of the study was to reproduce available experimental findings about the relationship between neuronal activity and cellular energy metabolism, while providing quantitative insights into the neuron-astrocyte functional interactions. We were particularly interested in establishing what triggers the activation of astrocytes during neuronal activity. Indeed, these cells are known to exhibit elevated rates of cerebral oxidative metabolism [15] that cannot be explained by the sole glutamate cycling [16]. In particular, the currently accepted estimates of brain energy utilization for signaling assign to astrocytes only glutamate-related energy use, which represents about 5 % of the total energy budget [1, 17]. However, a number of experimental ^13^C-MRS studies indicated that astrocytes account for up to 30 % of brain energy metabolism in anesthetized and awake rats (see Table 2 for references). The principal energy-demanding homeostatic function of astrocytes is reuptake of neuronally released K^+^ not glutamate (reviewed in [18]), as further evidenced by many studies in cultured astrocytes that have demonstrated that metabolism of these cells is stimulated by K^+^ rather than glutamate [19–23]. Besides being energetically expensive, astrocytic K^+^ uptake is tightly linked to neuronal activity, because most K^+^ is released into extracellular space by neuronal voltage/ligand-gated K^+^ channels. Importantly, glutamate reuptake by astrocytes only takes place in perisynaptic astrocytic processes, as glutamate spillover from synaptic cleft is spatially restricted (e.g., [24]). On the contrary, neuronal K^+^ release occurs along the length of axons and dendrites and it is thus capable of effectively activating periaxonal and peridendritic peripheral astrocytic processes (>60 % of astrocyte surface area). These notions suggest that the functional and metabolic interactions between neurons and astrocytes might involve primarily K^+^ [25].

A recent paper by Leif Hertz and colleagues suggested that astrocytic activation might be induced by the stoichiometry underlying the action of cellular Na^+^/K^+^ ATPase (NKA), which unevenly transports Na^+^ and K^+^ across plasma membrane according to a 3/2 ratio [26]. This idea is supported by the facts that (a) during neuronal activity K^+^ exits neurons in amounts comparable to the Na^+^ entering them [26, 27] and (b) astrocytic but not neuronal NKA is stimulated by excess extracellular K^+^ [28–30]. Here we relied on the assumption that neuronal glutamatergic neurotransmission is associated to voltage/ligand-gated ionic currents. This assumption is supported by the finding that, for example, there is proportionality between electroencephalographic signals (related to neuronal activity and therefore to ion movements) and the neurotransmitter cycling rate (which involves glutamate release) in the rat brain over a wide range of conditions (from isoelectric to near-resting levels) [31]. From the perspective of mass-balance modeling, the sole viable approach for implementing such association is to establish a stoichiometric relation between the two processes, keeping in mind that that such stoichiometry in principle might not exist explicitily, but may rather be “hidden” in other mechanisms that are indirectly related to ionic currents. The strategy is similar to what is commonly done for other lumped biochemical reactions (e.g., paracellular diffusion or yield in ATP by the respiratory chain) that are not stoichiometric per se. Therefore, our approach should be considered as a first account on the feasibility of incorporating activity-dependent ionic fluxes in stoichiometric models. Specifically, we tested whether signaling mechanisms in neurons, in terms of glutamate release and aggregated presynaptic and postsynaptic Na^+^ and K^+^ fluxes, can reproduce experimental observations of activity-induced stimulation of astrocytes in rat brain based on stoichiometry alone.

## Methods

We designed a compartmentalized metabolic network made up of blood capillary (b), extracellular space (e) and cellular compartments including neurons (n) and astrocytes (a). In the neuronal and astrocytic elements, we further distinguished cytosol (nc and ac), mitochondria (nm and am) and synaptic vesicles (nv, only in neurons). These cell compartments account for most of tissue volume (e.g., neurons 45–55 %, astrocytes 15–25 %, extracellular space 20 %). We neglected the contribution of GABAergic inhibitory interneurons, as excitatory glutamatergic neurons (85 % of total neurons) and protoplasmic astrocytes are the main cell types within the cortical grey matter of the brain [15]. The network incorporates the main reaction/transport processes of energy metabolism of carbohydrates in the brain. In addition, we modeled a stoichiometric relation between neuronal glutamate release and voltage/ligand-gated neuronal Na^+^ and K^+^ currents. Note that neither neuronal nor astrocytic NKA-catalyzed reactions were directly associated to the voltage-gated ion fluxes. As NKA is the principal activity-dependent determinant in ATP hydrolysis, our choice allows investigating the effect of the link between neuronal glutamatergic neurotransmission and associated ion currents on energy metabolism of neurons and astrocytes.

In all simulations, we kept the number of constraints (in addition to the stoichiometry of the metabolic network) to a bare minimum. In particular, we set the upper bound of glutamate-glutamine cycle (Vcyc, defined as J_a→e_SN, see Table 1) to Vcyc^0^ = 0.51 μmol g^−1^ min^−1^, which is the value obtained with ^13^C nuclear magnetic resonance spectroscopy (MRS) in awake rats [32]. Such a procedure is equivalent to introduce an arbitrary numeric scaling factor for the output fluxes, which in itself cannot be considered as a constraint. The sole hard constraint we introduced was the fraction of energy used by signaling versus that used by housekeeping functions. Specifically, we constrained the sum of neuronal plus astrocytic ATPase fluxes directed to housekeeping in the range 3–6 μmol g^−1^ min^−1^, or ~10–20 % of glucose utilization [33], assuming 32 ATP molecules produced per glucose metabolized and a cerebral metabolic rate of glucose of 0.91 μmol g^−1^ min^−1^ [32]. It is noted that at the whole-brain level (i.e. including white-matter), the fraction of energy allocated to housekeeping is somewhat greater, with different estimates giving values ranging from 25 % in rodents [16, 34] to 50 % in humans [35] (with the latter value likely overestimated, see [33] and references therein). Altogether, the metabolic network consists of 120 metabolites and 119 fluxes (Table 1). A brief description of the pathways included in the present model is provided in Online Resource 1. We also provide the model containing all reaction/transport fluxes in the System Biology Markup Language (SBML) format (Online Resource 2).

**Table 1:** Stoichiometry of reaction/transport fluxes constituting the metabolic network

Flux name	Stoichiometry	Enzyme/transporter/process
Glutamatergic activity (including neurotransmission, ionic movements and glutamate-glutamine cycle)		
J_n_NT	GLU_nv_ + a Na_e_ + b K_n_ → GLU_e_ + a Na_n_ + b K_e_	Glutamatergic neurotransmission GLU: glutamate Na: sodium K: potassium
J_e→a_EAAT	GLU_e_ + 3 Na_e_ + K_a_ → GLU_ac_ + 3 Na_a_ + K_e_	Excitatory amino acid transporter (EAAT)
J_n_VGLUT	GLU_nc_ + 1.5 ATP_n_ → GLU_nv_ + 1.5 ADP_n_	Vesicular glutamate transporter (VGLUT) ATP: adenosine triphosphate ADP: adenosine diphosphate
J_x_NKA	3 Na_x_ + 2 K_e_ + ATP_x_ → 3 Na_e_ + 2 K_x_ + ADP_x_	Neuronal/astrocytic Na/K-activated ATPase (NKA)
J_a→n_KIR	K_a_ → K_n_	K inward rectifying (KIR) channels
J_a_Nax	Na_e_ → Na_a_	Astrocytic Na-sensing Na channel (Nax)
J_a_GS	GLU_ac_ + ATP_a_ + NH_4,a_ → GLN_a_ + ADP_a_	Astrocytic glutamine synthetase (GS) NH_4_: ammonia GLN: glutamine
J_a→e_SN (V_cyc_)	GLN_a_ → GLN_e_	Astrocytic glutamine system N (SN) transporter
J_e→n_SA	GLN_e_ + Na_e_ → GLN_n_ + Na_n_	Neuronal glutamine system A (SA) transporter
J_n_PAG	GLN_n_ → GLU_nc_ + NH_4,n_	Neuronal phosphate-activated glutaminase (PAG)
Blood–brain nutrients (glucose/oxygen) transport		
J_b→e_GLUT	GLC_b_ → GLC_e_	Blood–brain glucose transporter (GLUT) GLC: glucose
J_e→x_GLUT	GLC_e_ → GLC_x_	Neuronal/astrocytic glucose transporter (GLUT)
J_→b_O_2_	→ O_2,b_	Blood oxygen diffusion (entry) O_2_: oxygen
J_b→x_O_2_	O_2,b_ → O_2,x_	Oxygen diffusion to neuron/astrocyte
J_x→b_CO_2_	CO_2,x_ → CO_2,b_	Carbon dioxide diffusion to blood CO_2_: carbon dioxide
J_b→_CO_2_	CO_2,b_ →	Blood carbon dioxide diffusion (exit)
Intercellular lactate trafficking		
J_x↔e_MCT	LAC_x_ ↔ LAC_e_	Neuronal/astrocytic monocarboxylate transporter (MCT) LAC: lactate
Glycolysis (Embden–Meyerhof–Parnas pathway)		
J_x_HK	GLC_x_ + ATP_x_ → G6P_x_ + ADP_x_	Neuronal/astrocytic hexokinase (HK) G6P: glucose 6-phosphate
J_x_PGI	G6P_x_ ↔ F6P_x_	Neuronal/astrocytic phosphoglucoisomerase (PGI) F6P: fructose 6-phosphate
J_x_PFK	F6P_x_ + ATP_x_ → FBP_x_ + ADP_x_	Neuronal/astrocytic phosphofructokinase (PFK) FBP: fructose 1,6-bisphosphate
J_x_ALD	FBP_x_ ↔ GAP_x_ + DHAP_x_	Neuronal/astrocytic aldolase (ALD) GAP: glyceraldehyde 3-phosphate DHAP: dihydroxyacetone phosphate
J_x_TPI	DHAP_x_ ↔ GAP_x_	Neuronal/astrocytic triosephosphate isomerase (TPI)
J_x_GAPDH	GAP_x_ + NAD_x_ ↔ BPG_x_ + NADH_x_	Neuronal/astrocytic glyceraldehyde 3-phosphate dehydrogenase (GAPDH) NAD(H): nicotinamide adenine dinucleotide BPG: 1,3-bisphosphoglycerate
J_x_PGK	BPG_x_ + ADP_x_ ↔ 3PG_x_ + ATP_x_	Neuronal/astrocytic phosphoglycerate kinase (PGK) 3PG: 3-phosphoglycerate
J_x_PGM	3PG_x_ ↔ 2PG_x_	Neuronal/astrocytic phosphoglycerate mutase (PGM) 2PG: 2-phosphoglycerate
J_x_ENO	2PG_x_ ↔ PEP_x_	Neuronal/astrocytic enolase (ENO) PEP: phosphoenolpyruvate
J_x_PK	PEP_x_ + ADP_x_ → PYR_x_ + ATP_x_	Neuronal/astrocytic pyruvate kinase (PK) PYR: pyruvate
J_x_LDH	PYR_x_ + NADH_x_ ↔ LAC_x_ + NAD_x_	Neuronal/astrocytic lactate dehydrogenase (LDH) LAC: lactate
Pyruvate recycling		
J_x_(c/m)ME	MAL_x(c/m)_ + NADP_x(c/m)_ → PYR_x(c/m)_ + NADPH_x(c/m)_	Neuronal/astrocytic cytosolic/mitochondrial malic enzyme (ME) MAL: malate NADP(H): nicotinamide adenine dinucleotide phosphate
Pyruvate carboxylation (anaplerosis)		
J_a_PC	PYR_am_ + ATP_a_ → OAA_am_ + ADP_a_	Astrocytic pyruvate carboxylase (PC) OAA: oxaloacetate
Tricarboxylic acid (TCA) cycle (Krebs cycle)		
J_x_mMCT	PYR_xc_ ↔ PYR_xm_	Neuronal/astrocytic mitochondrial monocarboxylate transporter (mMCT)
J_x_PDH	PYR_xm_ + CoA_x_ + NAD_xm_ → ACoA_x_ + CO_2,x_ + NADH_x_	Neuronal/astrocytic pyruvate dehydrogenase (PDH) CoA: coenzyme A ACoA: acetyl-coenzyme A
J_x_CS	OAA_xm_ + ACoA_x_ → CIT_x_ + CoA_x_	Neuronal/astrocytic citrate synthase (CS) CIT: citrate
J_x_ACO	CIT_x_ ↔ ISO_xm_	Neuronal/astrocytic mitochondrial aconitase (ACO) ISO: isocitrate
J_x_IDH1/2/3	(1) ISO_xm_ + NAD_xm_ → AKG_xm_ + CO_2,x_ + NADH_xm_ (2) ISO_xm_ + NADP_xm_ → AKG_xm_ + CO_2,x_ + NADPH_xm_ (3) ISO_xc_ + NADP_xc_ → AKG_xc_ + CO_2,x_ + NADPH_xc_	Neuronal/astrocytic cytosolic/mitochondrial isocitrate dehydrogenase (IDH) AKG: α-ketoglutarate (oxoglutarate)
J_x_AKGDH	AKG_xm_ + CoA_x_ + NAD_xm_ → SCoA_x_ + CO_2,x_ + NADH_xm_	Neuronal/astrocytic α-ketoglutarate dehydrogenase (AKGDH) SCoA: succinyl coenzyme A
J_x_SCS	SCoA_x_ + ADP_x_ ↔ SUC_x_ + CoA_x_ + ATP_x_	Neuronal/astrocytic succinyl coenzyme A synthetase (SCS) SUC: succinate
J_x_SDH	SUC_x_ + (2/3) NAD_xm_ ↔ FUM_x_ + (2/3) NADH_xm_	Neuronal/astrocytic succinate dehydrogenase (SDH) FUM: fumarate
J_x_FUM	FUM_x_ ↔ MAL_xm_	Neuronal/astrocytic fumarase (FUM)
J_x_(c/m)MDH	OAA_x(c/m)_ + NADH_x(c/m)_ → MAL_x(c/m)_ + NAD_x(c/m)_	Neuronal/astrocytic cytosolic/mitochondrial malate dehydrogenase (MDH)
Mitochondrial carriers		
J_x_GC	GLU_xc_ ↔ GLU_xm_	Neuronal/astrocytic mitochondrial glutamate carrier (GC)
J_x_DCC	MAL_xm_ ↔ MAL_xc_	Neuronal/astrocytic mitochondrial dicarboxylate carrier (DIC)
J_x_CIC	(1) ISO_xm_ ↔ ISO_xc_ (2) CIT_xm_ ↔ CIT_xc_	Neuronal/astrocytic mitochondrial citrate-isocitrate carrier (CIC)
Fatty acid synthesis (shunt)		
J_x_ACL	CIT_xc_ + ATP_x_ + CoA_x_ → OAA_xc_ + ADP_x_ + ACoA_x_	Neuronal/astrocytic ATP citrate lyase (ACL)
Mitochondrial NADH shuttles		
J_x_G3PS	NADH_xc_ + (2/3) NAD_xm_ → NAD_xc_ + (2/3) NADH_xm_	Neuronal/astrocytic glycerol 3-phosphate shuttle (G3PS)
J_x_OGC	MAL_xc_ + AKG_xm_ → MAL_xm_ + AKG_xc_	Neuronal/astrocytic oxoglutarate carrier (OGC)
J_x_AGC	GLU_xc_ + ASP_xm_ → GLU_xm_ + ASP_xc_	Neuronal/astrocytic aspartate-glutamate carrier (AGC) ASP: aspartate
J_x_(c/m)AAT	ASP_x(c/m)_ + AKG_x(c/m)_ ↔ OAA_x(c/m)_ + GLU_x(c/m)_	Neuronal/astrocytic cytosolic/mitochondrial aspartate aminotransferase (AAT)
Ammonia homeostasis		
J_x_GDH	GLU_xm_ + NAD_xm_ ↔ AKG_xm_ + NADH_xm_ + NH_4,x_	Neuronal/astrocytic glutamate dehydrogenase (GDH)
J_x_ALAT	GLU_xm_ + PYR_xm_ ↔ AKG_xm_ + ALA_x_	Neuronal/astrocytic alanine aminotransferase (ALAT) ALA: alanine
J_x↔e_ALA	ALA_x_ ↔ ALA_e_	Neuronal/astrocytic alanine transporter
J_x_BCAT	GLU_xm_ + BCKA_x_ ↔ AKG_xm_ + BCAA_x_	Neuronal/astrocytic branched-chain aminotransferase (cBCAT) BCKA: branched-chain keto acid BCAA: branched-chain amino acid
J_x↔e_BCAA	BCAA_x_ ↔ BCAA_e_	Neuronal/astrocytic branched-chain amino acid transporter
J_x↔e_BCKA	BCKA_x_ ↔ BCKA_e_	Neuronal/astrocytic branched-chain keto acid transporter
Oxidative phosphorylation		
J_x_OP	2 NADH_xm_ + 5 ADP_x_ + O_2,x_ → 2 NAD_xm_ + 5 ATP_x_ + c ROS_x_	Neuronal/astrocytic respiration ROS: reactive oxygen species
Housekeeping		
J_x_ATPase	ATP_x_ → ADP_x_	Neuronal/astrocytic ATPases (other than NKA)
Antioxidant system		
J_x_PPP	3 G6P_x_ + 6 NADP_xc_ → GAP_x_ + 2 F6P_x_ + 6 NADPH_xc_	Neuronal/astrocytic pentose phosphates pathway
J_x_(c/m)GR	GSSG_x_ + NADPH_x(c/m)_ → 2 GSH_x_ + NADP_x(c/m)_	Neuronal/astrocytic cytosolic/mitochondrial glutathione reductase (GR) GSSG: glutathione disulfide GSH: glutathione
J_x_GPX	2 GSH_x_ + ROS_x_ → GSSG_x_	Neuronal/astrocytic glutathione peroxidase

Subscripts indicate tissue compartments (see text). Subscript x indicates either n (neuronal) or a (astrocytic) compartment

In the stoichiometry equations the single and double arrows represent irreversible and reversible fluxes, respectively

Optimized stoichiometric coefficients for NKA-catalyzed reactions are a = 63 and b = 58. Optimized stoichiometric coefficient for ROS generation in oxidative phosphorylation are c = 0.025 (neurons) and c = 0.250 (astrocytes)

The sampling algorithm used to obtain distribution of fluxes was developed employing constraint logic programming (CLP) over real numbers (CLPℜ) [36]. The details of the CLP-based sampling algorithm implemented to solve flux balance analysis (FBA) problems, as well as convergence diagnostics and benchmarking against the Artificial Centering Hit-and-Run algorithm, are given in Online Resource 3. Briefly, given a set of variables (fluxes) and constraints (stoichiometry), the algorithm iteratively binds a variable (randomly from the set of variables) to a value (randomly from the variable domain). This binding represent a further constraint, which eventually reduces the domains of the other unbound variables [37]. If these additional constraints result in inconsistent domain for some unbound variables, then the algorithm restarts with all unbounds variables, otherwise the solution is valid and it is recorded. The algorithm was implemented under SWI Prolog (University of Amsterdam, The Netherlands; http://www.swi-prolog.org) running the built-in CLPℜ library. Results of the simulations were analyzed using MATLAB (The Mathworks Inc., Natick, MA, USA; http://www.mathworks.com/).

## Results

We first determined the initial domains of the fluxes underlying our metabolic network (see Online Resource 4). The pruning of solution space brought about by CLP is remarkable, with most fluxes constrained to values below 5 μmol g^−1^ min^−1^ and maximal range of about 12 μmol g^−1^ min^−1^. It should be noted that the actual solutions (i.e. the distributions obtained through sampling) will map a subset of the variable domains depending on the specified constraints.

**Fig. 1:**
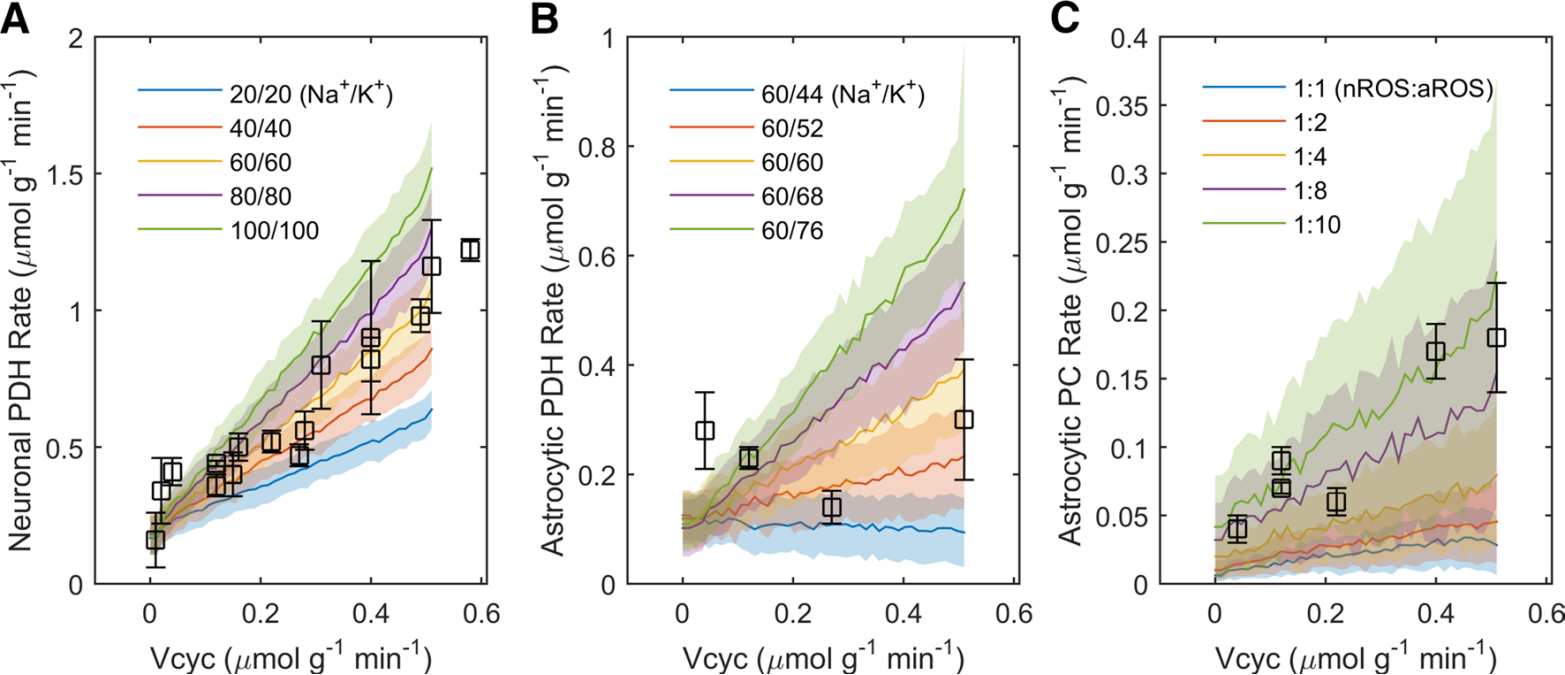
Validation of the model against experimental data. The agreement between simulations and specific experimentally measured fluxes has been examined at the stoichiometric level (i.e. without introducing constraints in addition to the metabolic network). **a** Neuronal PDH rate is controlled by the absolute amount of Na^+^ (K^+^) ions entering (exiting) the cell per transmitter released, here always in a 1:1 ratio. The optmized value is 63 ions moving per each glutamate molecule. **b** Astrocytic PDH rate is controlled by the imbalance between neuronal voltage/ligand-gated Na^+^ and K^+^ ionic currents. The optimized proportion is 58 K^+^ exiting neurons during concomitant entry of 63 Na^+^ per each glutamate molecule. Note that neuronal and astrocytic PDH rates depends on the energy expended by NKA to move Na^+^ and K^+^ in opposite direction relative to depolarization/hyperpolarization underlying neuronal activity. While neuronal metabolism is sensitive to the magnitude of Na^+^ influx/K^+^ efflux, astrocytic metabolism responds to the imbalance between those fluxes. **c** Astrocytic PC rate is controlled by the relative rate of ROS production in astrocytes compared with neurons. The optimized value is a tenfold higher ROS production in astrocytes relative to neurons. Simulated values are expressed as mean ± SD. Experimental data points are listed in Table 2

**Fig. 2:**
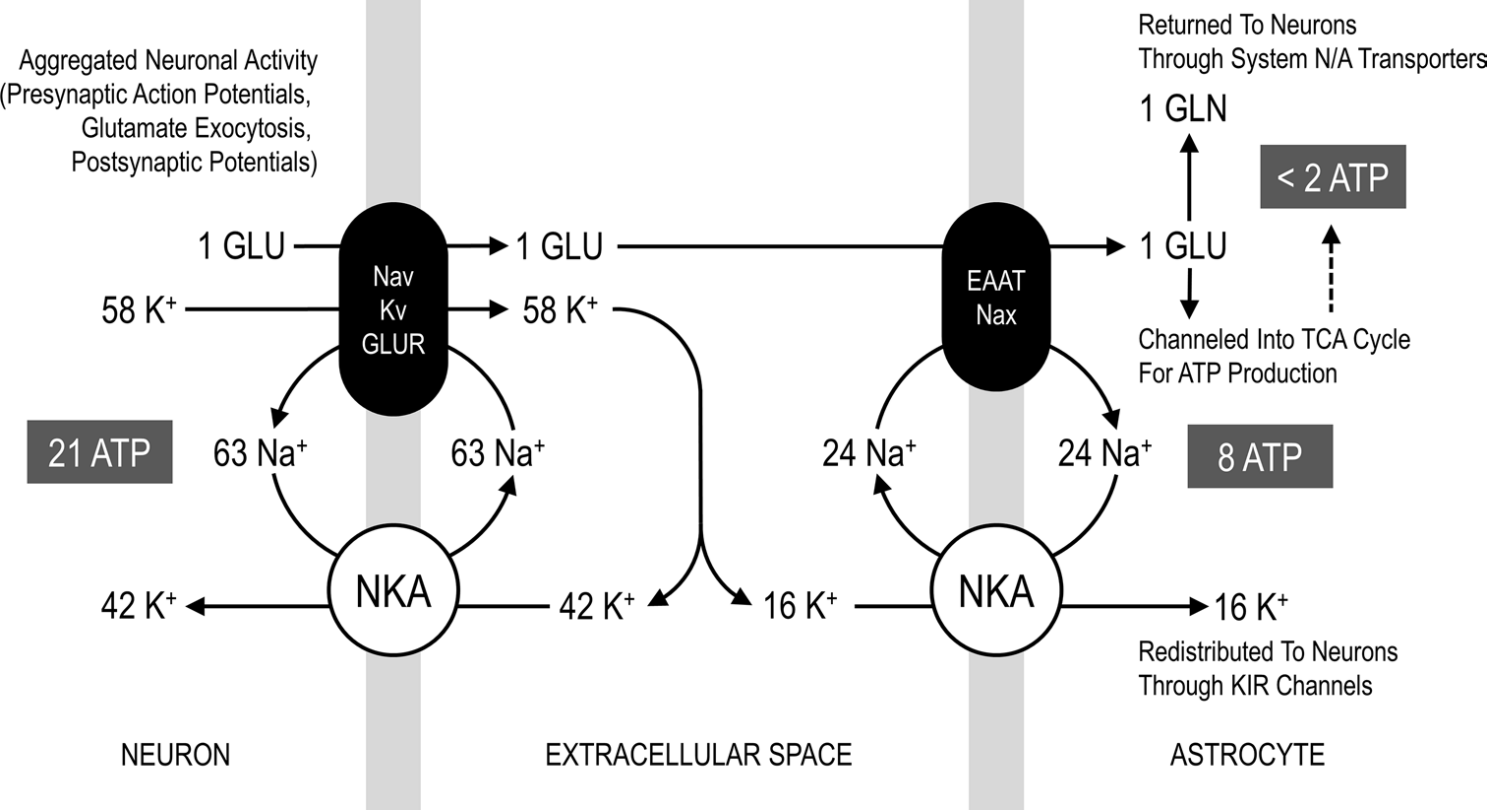
Stoichiometry for voltage/ligand-gated Na^+^ and K^+^ channel currents associated with neuronal glutamatergic neurotransmission. Simplified schematics of the stoichiometric relation between the exocytosis of 1 glutamate molecule, influx of 63 Na^+^ ions (i.e. depolarization) and efflux of 58 K^+^ ions (i.e. repolarization). These values are the results of the fitting procedure between model outcomes and experimental ^13^C-MRS obtained in rat brain during different activity levels (see Fig. 1). The corresponding ratio between Na^+^ and K^+^ fluxes due to neuronal activity is thus close to 1:1. Yet, NKA (both neuronal and astrocytic) works in a 3/2 Na^+^/K^+^ ratio. Therefore, only part of neuronally released K^+^ (42/58, i.e. around 72 %) is taken up directly by these cells, with concomitant hydrolysis of 21 ATP molecules. The difference in the magnitude of ionic currents underlying neuronal activity and the opposite ionic movements underlying NKA action in neurons results in an excess of 16 K^+^ ions in extracellular space, which are actively taken up by astrocytes and passively redistributed to neurons. Overall, the astrocytic uptake of the fraction of neuronally released K^+^ (16/58, i.e. around 28 %) is associated with the hydrolysis of 8 ATP molecules. At the same time, the glutamate molecule is taken up and converted to glutamine, with an associated hydrolysis of 1–2 ATP molecules (notice that glutamate reuptake is also associated with corelease of 1 K^+^, not shown in the figure). However, the latter number is also reduced by the fact that some glutamate is channeled into TCA cycle, thereby providing ATP for its own uptake. The above-mentioned processes provide the following energy budget for signaling (i.e. excluding housekeeping): nearly 67.5 % of energy is used by neuronal glutamatergic activity, 26 % by astrocytic K^+^ reuptake and 6.5 % for glutamate recycling. Astrocytic energy expenditure accounts for one-third of the total, most of which (~80 %) is devoted to active K^+^ reuptake. Please note that Na^+^ cycle in astrocytes can be supported by many pathways other than the extracellular Na^+^-sensitive Na^+^ channel (Nax) and excitatory amino acid transporter (EAAT) proteins (see Online Resource 1)

The stoichiometry of the metabolic network is almost completely determined by biochemical reaction/transport processes. However, the quantitative mass-balance underlying the relation between glutamate release and ionic movements is unknown. Based on biophysical calculations it has been estimated that about 400,000 Na^+^ ions enter neurons through voltage/ligand-gated channels (i.e. aggregated for presynaptic and postsynaptic currents) per each glutamate vesicle released [16]. Assuming an average vesicular content in the range of 2000–6000 glutamate molecules [38–41] gives a flux of 60–200 Na^+^ ions per glutamate molecule. We found that a value around the low-end of 60 Na^+^ per glutamate (for sodium influx and also for potassium efflux, see below) correctly reproduces the experimental relationship between the rates of glutamate-glutamine cycle and neuronal oxidative metabolism (Fig. 1a). The Na^+^ influx and K^+^ efflux underlying neuronal signaling are thought to be of comparable magnitude. However, the (electrogenic) NKA responsible of restoring ion concentrations operates with a 3/2 Na^+^/K^+^ flux ratio. The resulting imbalance between Na^+^ and K^+^ ionic movements (inward Na^+^/outward K^+^ due to neuronal activity and outward Na^+^/inward K^+^ due to NKA) produces an excess K^+^ in the extracellular space, which is likely removed by astrocytes [26]. Our model fully supports this notion. Simulations performed with various neuronal voltage/ligand-gated Na^+^/K^+^ influx/efflux ratios largely determined the degree of astrocytic activation, which is completely abolished when the ratio is close to 3/2 (Fig. 1b). Overall, using a 63/58 Na^+^/K^+^ ratio for neuronal ionic fluxes (Fig. 2), the cellular rates of metabolic pathways for oxidative metabolism of glucose are consistent with experimental data (Fig. 1; Table 2).

**Table 2:** Metabolic fluxes measured with ^13^C-MRS in adult rat gray matter under different activity levels

References	Vcyc	J_n_PDH	J_a_PDH	J_a_PC
Deep anesthesia (isoelectric EEG)				
[42]	0.01 ± 0.01	0.16 ± 0.10	–	–
[43]	0.02 ± 0.01	0.34 ± 0.12	–	–
[44]	0.04 ± 0.01	0.41 ± 0.05	0.28 ± 0.07	0.04 ± 0.01
Moderate anesthesia				
[42]	0.15 ± 0.05	0.40 ± 0.08	–	–
[45]	0.12 ± 0.01	0.44 ± 0.01	0.23 ± 0.02	0.070 ± 0.004
[46, 47]	0.22 ± 0.04	0.52 ± 0.04	–	0.06 ± 0.01^a^
[48]	0.12 ± 0.01^c^	0.36 ± 0.04^c^	–	0.09 ± 0.01^d^
[49]	0.27 ± 0.02	0.47 ± 0.04	0.14 ± 0.03	–
[50]	0.16 ± 0.04	0.50 ± 0.05	–	–
Light anesthesia				
[51]	0.28 ± 0.03	0.56 ± 0.07	–	–
[48]	0.40 ± 0.04^c^	0.82 ± 0.08^c^	–	0.17 ± 0.02^d^
[42]	0.40 ± 0.13	0.90 ± 0.28	–	–
[52]	0.31 ± 0.17	0.80 ± 0.16	–	–
[43]	0.58 ± 0.02	1.22 ± 0.04	–	–
Awake				
[53]	0.49 ± 0.05^b^	0.98 ± 0.06^b^	–	–
[32]	0.51 ± 0.21	1.16 ± 0.17	0.30 ± 0.11	0.18 ± 0.04

All values are expressed in units µmol g^−1^ min^−1^ (mean ± SD). Approximate state of the animals range from deep, moderate, light anesthesia to awake, as indicated. These conditions are associated with the different metabolic rates plotted in Fig. 1. For details about experimental conditions (e.g., employed anesthetics) see individual studies. Some data appear as they were reanalyzed elsewhere [33, 54]

^a^Estimated based on identical values of neuronal PDH in two different studies under the same experimental conditions

^b^Averaged from frontal, parietal, temporal and occipital cortex

^c^Error estimated as 10 % of the experimental measurement, which corresponds to the average error of tabulated studies

^d^Calculated from measured ratio between PC and neuronal PDH

We found that the experimentally observed rate of pyruvate carboxylation at Vcyc = Vcyc^0^ = 0.51 μmol g^−1^ min^−^1 (awake value) is obtained by assuming that astrocytic production of ROS per oxygen consumed is tenfold higher than neurons (Fig. 1c), which possibly reflects a lower activity/expression of mitochondrial bound kinases, as previously suggested [18]. As expected, ROS generation affects the rate of PPP, which turns out to be several-fold higher in astrocytes compared with neurons (8–11 nmol g^−1^ min^−1^ vs. 2 nmol g^−1^ min^−1^, see Online Resource 10 Panels N,T), which is consistent with current literature ([55] and references therein). The activity-dependent increase in PC rate due to ROS scavenging is brought about by the regeneration of NADPH through pyruvate-malate shuttle. This pathway makes PC activity independent of any carbon loss from the astrocytic TCA cycle (see below). It is noted that other mechanisms might participate in stimulating astrocytic PC rate, such as glutamine and/or citrate efflux (see Online Resource 1), that could be fine-tuned to obtain similar model outcomes.

**Fig. 3:**
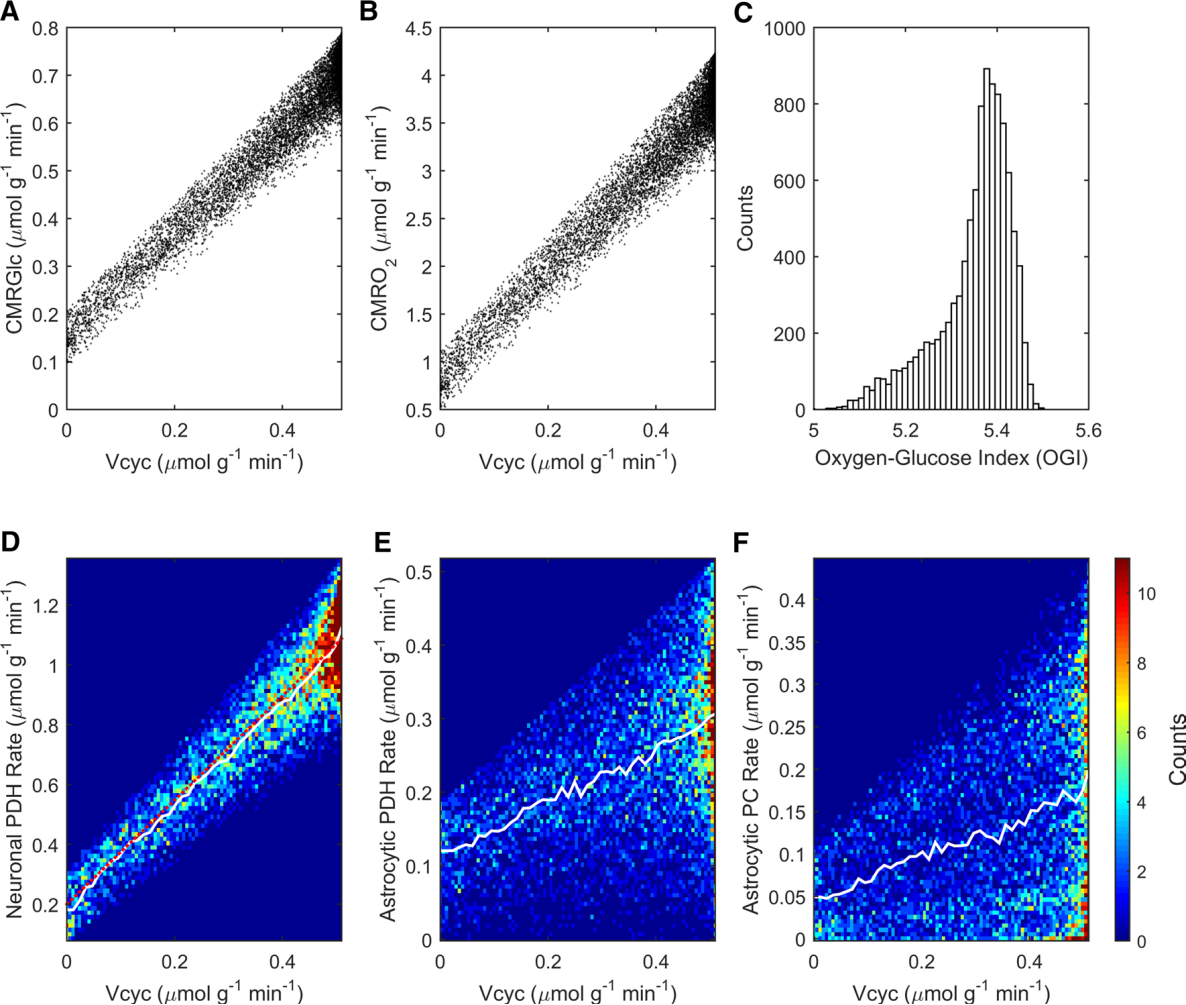
Model outcomes for tissue and cellular oxidative metabolism. The simulated fluxes of cerebral metabolic rate of **a** glucose (CMRGlc) and **b** oxygen (CMRO_2_) are associated with a range of **c** oxygen-to-glucose index (OGI) that is consistent with the reported values measured experimentally. **d–f** Results of optimization of stoichiometric coefficients (see Table 1; Fig. 1) for neuronal PDH, astrocytic PDH and astrocytic PC, respectively. Counts in different panels refer to the actual number of solutions falling in a given bin (either color-coded or as histogram), which sum up to 10,000. *Color plots* have been obtained by computing the histograms of solutions (bins number determined according to Rice rule, or 2*n* ^1/3^, where *n* is the total number of solutions). Note that the upper bound of the abscissa in panels **a, b, d, e, f** is at Vcyc = Vcyc^0^ = 0.51 μmol g^−1^ min^−^1 (awake value). (Color figure online)

Model results are consistent with the experimentally measured rates of both cerebral glucose (Fig. 3a) and oxygen (Fig. 3b) utilization and associated range of oxygen-to-glucose index (OGI) (Fig. 3c). Simulated values for cell oxidative metabolism are in very good agreeement with experimental ^13^C-MRS data. In particular, the simulated reaction rates of neuronal (Fig. 3d) and astrocytic (Fig. 3e) pyruvate dehydrogenase as well as the rate of astrocytic pyruvate carboxylase (Fig. 3f) at Vcyc = Vcyc^0^ match on average the experimental values of 1.16, 0.30 and 0.18 μmol g^−1^ min^−1^ measured under awake conditions in rat brain [32], respectively.

**Fig. 4:**
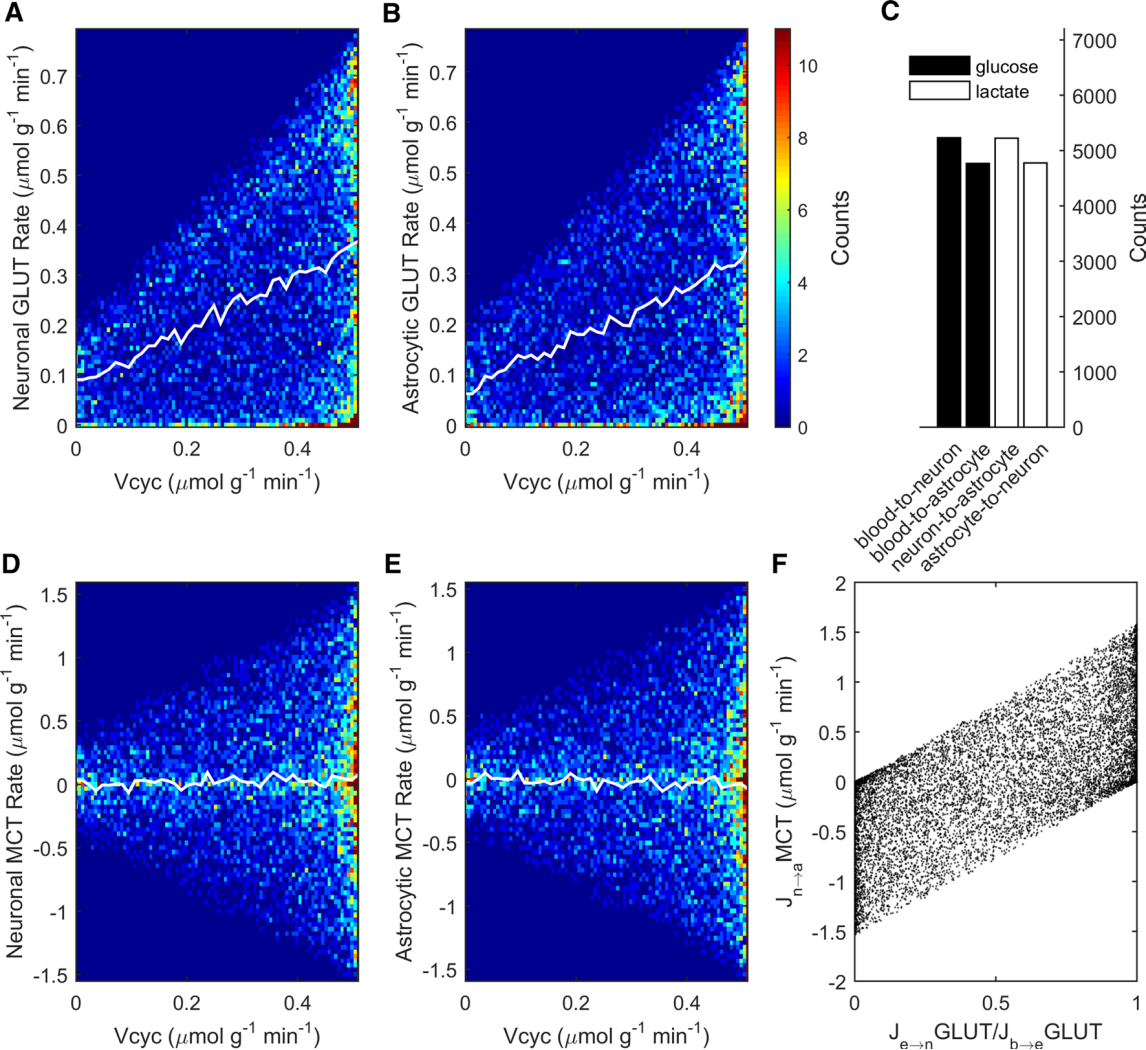
Model outcomes for cellular glucose uptake and lactate shuttle. Simulated glucose uptake by **a** neuronal and **b** astrocytic GLUTs and concomitant lactate transport by **d** neuronal and **e** astrocytic MCTs. On average, glucose is taken up almost equally by neurons and astrocytes and intercellular lactate transfer is negligible across all activation levels. However, feasible solutions include extreme cases with one cell type taking up all glucose and the other relying exclusively on shuttled lactate. **c** The number of solutions supporting predominant neuronal glucose uptake and lactate release are slightly more numerous than solutions supporting the opposite. **f** The direction and magnitude of cell-to-cell lactate shuttle is directly controlled by the cellular uptake of glucose. Note that in order to compare the GLUT and MCT fluxes in terms of glucose equivalents, the MCT flux has to be divided by two. *Color plots* have been obtained as in Fig. 3. Note that the upper bound of the abscissa in panels **a, b, d, e** is at Vcyc = Vcyc^0^ = 0.51 μmol g^−1^ min^−^1 (awake value). (Color figure online)

Under the conditions that reproduced the aforementioned experimental results, simulations showed that glucose is on average almost equally taken up by neurons and astrocytes (Fig. 4a, b) while intercellular lactate trafficking is negligible (Fig. 4d, e). In fact, there are slightly more solutions supporting a predominant neuronal glucose uptake and associated lactate release (Fig. 4c). The preference for a small lactate transfer (0.05 μmol g^−1^ min^−1^) from neurons to astrocytes under awake conditions supports recent modeling studies [56, 57]. However, there is no apparent correlation between intercellular lactate trafficking and neurotransmission level, in agreement with our previous theoretical analysis [6]. In fact, it is the exact value of glucose partitioning between the two cell types to completely determine the direction and magnitude of lactate transfer (Fig. 4f), as previously reported [5, 6]. On average, simulations show that under awake conditions neurons and astrocytes take up a similar fraction of glucose (~0.35 μmol g^−1^ min^−1^, respectively).

**Fig. 5:**
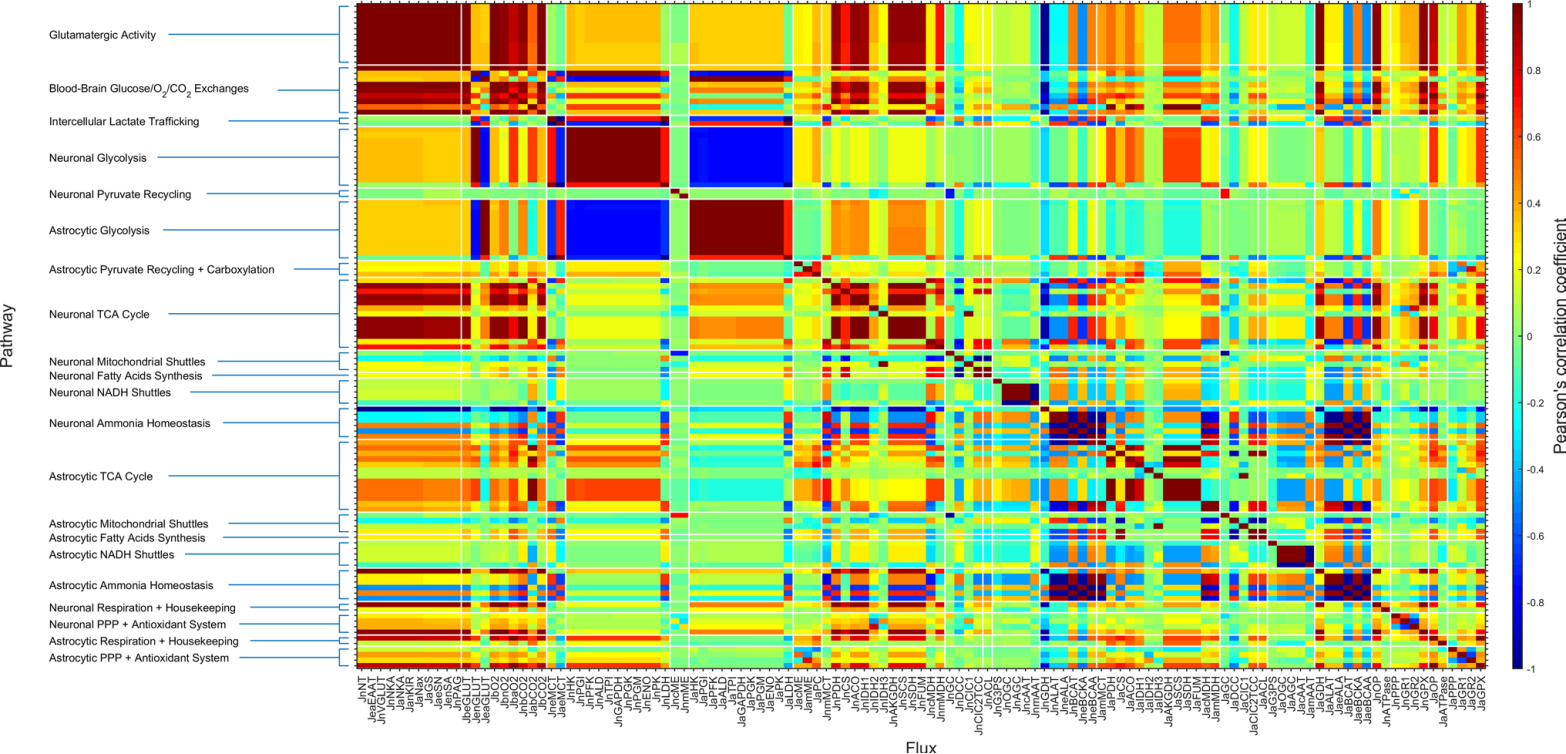
Spread matrix of model solutions. Flux names are reported at the *bottom*, while grouped metabolic pathways are reported *sideways*. There is strong correlation between the reactions within the “glutamatergic activity” group as well as between this group and metabolic pathways (glycolysis, TCA cycle, respiration). As expected, pathways in the “ammonia homeostasis” group (including glutamate dehydrogenase-catalyzed reaction) exhibit opposite correlations in neurons and astrocytes. Cellular glycolysis and TCA cycle also form correlated groups. Noticeably, neuronal glycolysis is more correlated with astrocytic rather than neuronal TCA cycle, and vice versa. The spread matrix has been determined by computing the Pearson’s correlation coefficient between each pair of reactions

**Fig. 6:**
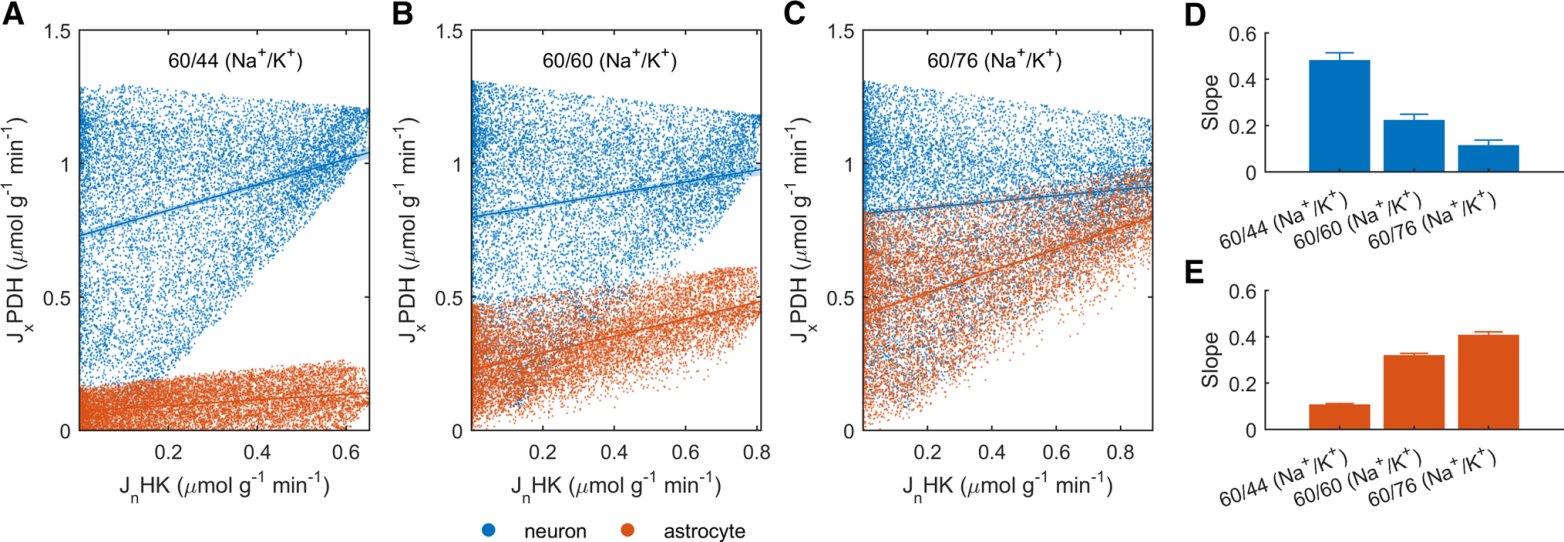
Dependence of neuronal glycolysis and astrocytic glycolysis/TCA cycle on the Na^+^/K^+^ fluxes ratio. In neurons, the rate of HK, i.e. the enzyme commiting glucose to cellular glycolysis, is positively correlated with the rate of PDH, i.e. the enzyme channeling the product of glycolytic pathway pyruvate to cellular TCA cycle. However, this positive correlation decreases with increasing departures in Na^+^ influx/K^+^ efflux ratio (due to neuronal activity) above the 3/2 ratio underlying the activity of NKA, from 60/44 (**a**) to 60/60 (**b**) and beyond to 60/76 (**c**). Concomitantly, when voltage/ligand-gated fluxes are above the 3/2 Na^+^/K^+^ NKA ratio, neuronal HK becomes more and more correlated with astrocytic PDH, reflecting the fact that excess K^+^ stimulates astrocytic activation and oxidative metabolism. Note that while the Na^+^ influx/K^+^ efflux ratio decreases, neuronally released K^+^ increases because Na^+^ influx is kept constant. **d, e** Summary of the slope of the linear curves fitting the simulated data. *Error bars* are determined by calculating the confidence intervals in each data plot with *p* < 0.001

In order to examine how the participation of astrocytes in neuronal activity affects the metabolic network of both cell types, we performed correlation analysis of fluxes (Fig. 5). The spread matrix shows that cellular glycolysis is strongly correlated within each individual cell types and strongly anticorrelated with the other cell type. Interestingly, the opposite is true, although less firmly, for cellular TCA cycle. In other words, there is a positive correlation between neuronal glycolysis and astrocytic TCA cycle, and vice versa. Interestingly, this behavior depends on the amount of neuronally released K^+^ (due to voltage/ligand-gated channels) in excess of the 3/2 Na^+^/K^+^ NKA ratio (Fig. 6). These results (and in particular, the loss of intercellular correlation) are obtained even in the presence of glutamatergic neurotransmission and glutamate-glutamine cycle, supporting the idea that astrocytic K^+^ uptake is the major mechanism underlying the metabolic relationship between neurons and astrocyte. This finding is further strengthened by the fact that blocking extracellular glutamate reuptake by astrocytes (and concomitantly allowing glutamate uptake directly by neurons) does not alter the correlation patterns between glycolysis and TCA cycle (Online Resource 5).

Finally, we compared flux distributions and corresponding average values obtained for the whole range of neuronal activity levels (0 ≤ Vcyc ≤ Vcyc^0^; Vcyc^0^ = 0.51 μmol g^−1^ min^−1^) with those obtained for the sole awake conditions (Vcyc = Vcyc^0^) (Online Resources 6–10 and 11–15, respectively). As expected, the distributions of fluxes change under awake conditions, confirming that fluxes are modulated by neuronal activity. However, there are certain fluxes exhibiting nearly the same distribution regardless of the activation state. These activity-independent fluxes include MCT (Online Resource 6 Panels U,V; compare with Online Resource 11 Panels U,V) and LDH (Online Resource 7 Panels I,V; compare with Online Resource 12 Panels I,V). This result indicates that cell-specific lactate production/utilization and cell-to-cell lactate shuttling are not constrained in any direction by the activity levels, as further support to previous modeling study [6]. As expected, the metabolic fluxes whose domains collapse into a single value (0.51 μmol g^−1^ min^−1^) when Vcyc = Vcyc^0^ are those involved in glutamate-glutamine cycle (Online Resource 6 Panels H,K; compare with Online Resource 11 Panels H,K) and associated ammonia homeostasis (Online Resources 9 Panel A and Online Resource 10 Panel F; compare with Online Resource 14 Panel A and Online Resource 15 Panel F). The distribution of fluxes for the whole metabolic network are provided as evidence for the good performance of our sampling algorithm. The archives containing the complete datasets for unconstrained and awake conditions are provided in Online Resource 16.

## Discussion

In the last years, novel thoughts have stimulated revisions of long-established notions about the metabolic pathways used to sustain brain function. An intense area of research has focused on the participation of astrocytes in virtually any aspects of neuronal signaling. In the cortical gray matter of the brain, these cells constitute nearly 20 % of brain tissue volume and account for up to 30 % of oxidative metabolism [15]. These relatively high metabolic rates, which per volume are comparable to those of neurons, could not be reproduced by theoretical studies that based astrocytic energy demand exclusively on recycling of transmitter glutamate (e.g., [16]). As we illustrated in the introduction, this latter assumption has contributed to promote the wrong notion that cortical astrocytes consume only few % of the brain’s energy budget. In recent years, it has become more and more clear that astrocytes are far more sensitive to increases in extracellular K^+^ than glutamate (reviewed by [18]). This means that any quantitative model of neuron-astrocyte interactions must take into account the contribution of astrocytes in the energy-requiring process of K^+^ uptake. Due to the difficulty of determing this contribution quantitatively, many mathematical models neglected tout-court astrocytic involvement in ion homeostasis and correspondingly underestimated activation of astrocytes and associated energy consumption. Here, we took advantage of experimental results obtained with ^13^C-MRS in rat brain, and determined a simple stoichiometric relation between glutamatergic neurotransmission and voltage/ligand-gated Na^+^ and K^+^ ion fluxes in neurons. We found that the 63/58 Na^+^ influx/K^+^ efflux per glutamate molecule underlying neuronal activity (see Fig. 1) is sufficient to reproduce the experimental measurements of major neuronal and, notably, astrocytic metabolic pathways (Figs. 2, 3, 4). In particular, the imbalance between the above-mentioned activity-dependent ion movements ratio (close to 1:1) and the 3/2 outward Na^+^/inward K^+^ NKA ratio can itself explain the finding that astrocytes account for nearly one-third of total brain energy metabolism.

Our results indicate that the experimental relationship between glutamate-glutamine cycle (Vcyc) and cellular oxidative metabolism of glucose (i.e. PDH reaction rate) is dictated for neurons by the absolute amount of neuronal Na^+^ and K^+^ ionic fluxes (Fig. 2a), and for astrocytes by the imbalance between these fluxes (Fig. 2b). The rate of astrocytic PC is instead not directly related to ionic movements. The choice to avoid assumptions on the loss of TCA cycle intermediates resulted in the finding that PC is only indirectly correlated to neuronal activity through the activity-dependent increase in ROS scavenging (Fig. 2c). The biochemical mechanism for increased PC rate and antioxidant system is the replenishment of the NADPH required by glutathione reductase. Specifically, combined action of malic enzyme and PC regenerates NADPH with concomitant hydrolysis of ATP. These reactions (pyruvate-malate shuttle) bypass the NADH-yielding malate dehydrogenase step of TCA cycle, which converts malate to oxaloacetate. Notably, PC activity becomes negatively correlated with PPP in astrocytes (Fig. 5), the other NADPH-producing pathway. However, the negative correlation between PC and PPP does not necessarily mean that PC is the primary mechanism for detoxification of ROS. Indeed, the model supports the notion that NAPDH is regenerated through PPP in an activity-dependent manner, as evidenced by the positive correlation between PPP and neuronal ionic currents and neurotransmission (Fig. 5). In other words, while PC and PPP are negatively correlated to each other, both are positively correlated with glutamatergic activity. The above-mentioned scenario depends on our choice to avoid assumptions about any activity-dependent loss of astrocytic TCA cycle intermediates (see Online Resource 1), as the exact biochemical mechanisms underlying the change in PC rate associated with activity levels remain to be experimentally established.

Overall, it is the departure of neuronal voltage/ligand-gated Na^+^ and K^+^ fluxes from the Na^+^/K^+^ NKA-dependent fluxes (1:1 for the former and 3/2 for the latter) that is primarily responsible for the establishment of the link between neuronal and astrocytic metabolism (Fig. 6). In particular, at increasing rates of K^+^ release relative to Na^+^, the neuronal glucose utilization (determined by HK reaction rate) becomes more and more correlated with astrocytic oxidative metabolism (determined by PDH reaction rate). This finding is consitent with the rise in astrocytic respiration induced by K^+^ reported experimentally (see [15]). We found that glutamate, which is commonly thought to represent the primary signal linking neuronal and astrocytic functional metabolism, does not appreciably affect the correlation patterns between cellular metabolic pathways (Online Resource 5). We also found that K^+^ uptake in astrocytes is necessary for the activity-dependent upregulation of energy metabolism in these cells, which is evidenced by the finding that under certain conditions astrocytic metabolism remains low even when glutamate-glutamine cycle increases (e.g., see Fig. 1b). Indeed, glutamate uptake by astrocytes requires much less energy than K^+^. According to our estimates, nearly 16 K^+^ ions (Fig. 2) enter astrocytes per each glutamate, thus requiring 8 ATP molecules (as NKA takes up 2 K^+^ per ATP hydrolyzed). Since the cycling of one glutamate molecule by astrocytes requires hydrolysis of two ATP molecules (reuptake plus conversion to glutamine), this means that overall energy consumption in astrocytes due to K^+^ uptake is about fourfold higher than that required by uptake of glutamate. Furthermore, glutamate but not K^+^ can be used as energy substrate to fuel its own uptake, which can even lower the energetic burden of astrocytes for glutamate uptake [58, 59].

The model upholds the concept that neurons and astrocytes can use whatever proportion of glucose and lactate (Fig. 4). Depending on cellular glucose uptake, lactate transport across cells is indeed adjusted to satisfy neuronal and astrocytic energy needs, as previously reported [3]. In particular, the stoichiometry is compatible with model solutions identifying one cell type as the sole compartment of glucose uptake and lactate release and the other cell type relying completely on lactate uptake. Since we decided to avoid the introduction of additional constraints to restrict the space of feasible solutions, we interpret such extreme cases as reflecting a lack of information in the present model (e.g. incomplete metabolic network and/or absence of regulatory mechanisms). In fact, these cases with poor physiological significance are canceled out by averaging across solutions. The distributions-based approach provides complementary information to objective-function optimization (see also [5, 8]), although further research is required to measure and interpret the rate of specific metabolic pathways in an activation-dependent manner. In agreement with previous works, our model outcomes support a lactate transfer from neurons to astrocytes [56, 57, 60]. We found that the contribution of this lactate shuttle is very small, and occurs on top of neuronal and astrocytic glucose uptakes which are similar to each other. Interestingly, recent experimental findings showed higher glucose uptake in neurons relative to astrocytes [61, 62], which suggests that astrocytic energy requirements are met by substrates other than blood-derived glucose. Glycogen is one major candidate in providing substrates for astrocytic metabolism, as K^+^ uptake in astrocytes is probably fueled by glycogenolysis [18, 63]. Interpretation of studies reporting predominantly astrocytic [64, 65] glucose uptake requires taking into account the impact of glycogen metabolism. Indeed, increased astrocytic glucose uptake observed in anesthesized animals [65] or tissue slices [64] might reflect inactive glycogenolysis due to anesthesia or tissue glycogen depletion, respectively [66]. Furthermore, the use of the glucose analogues used in the studies that have reported higher astrocytic versus neuronal glucose uptake [64, 65] has been criticized based on transport kinetic analysis [67]. Finally, glucose utilization in astrocytes may be biased in the ^13^C-MRS studies of anesthesized animals due to anesthesia-induced decrease in glycogen degradation and/or increase in glycogen synthesis. Unfortunately, glycogen metabolism cannot be incorporated into mass-balance models like the present one, unless ad-hoc constraints about the rate of glycogenolysis (currently unknown) are introduced.

One main limitation of the present FBA-based model is that metabolism can be studied only under steady-state conditions. Theoretical investigations about the transient metabolic processes (e.g., glycogenolysis) occurring in response to physiological stimulation on top of the awake condition require the development of kinetic models [60, 68]. This is especially relevant in the short-term response of the cortical tissue to stimulation, which is confined to tens of seconds/few minutes before a new, activated steady-state is eventually established [69, 70]. Another potential limitation is that we assumed a fixed stoichiometry for ionic currents induced by glutamatergic signaling across all activity levels, from isoelectric to awake conditions. In particular, the quantitative relation between synaptic and spiking activity might produce dynamic ion movements across neurons and astrocytes in an activity-dependent manner, although such non-linear behavior is likely to be of relevance only at high physiological activity (i.e. under intense stimulation) [2]. Furthermore, it should be noted that the stoichiometry of 63 Na^+^ entering and 58 K^+^ exiting neurons per glutamate released comes from the assumption of a direct quantitative link between glutamatergic neurotransmission and voltage/ligand-gated ionic currents. In fact, other mechanisms may indirectly concur to define such stoichiometry. For example, part of the inward neuronal Na^+^ current can be carried by Ca^2+^ through the action of Na^+^/Ca^2+^ exchanger [16]. Nonetheless, our model provides a theoretical framework to test various hypotheses about the relevance of specific metabolic pathways. For example, targeted enzyme deletions might be used to identify reaction/transport processes that are involved and/or essential in order to reproduce or support a certain experimental result. In this respect, a preliminary implication of our results is that under awake conditions glutamate dehydrogenase (GDH) appears to be necessary for neurotransmission (Online Resource 10 Panel F and Online Resource 15 Panel F). Indeed, glutamate-glutamine cycle entails concurrent maintenance of ammonia homeostasis in neurons and astrocytes, which requires GDH regardless of the identity of the associated amino acid shuttle [71]. Our results support the notion that GDH-catalyzed reaction runs in opposite direction in these cell types, aminating α-ketoglutarate in neurons and deaminating glutamate in astrocytes, thereby removing (in neurons) or providing (in astrocytes) ammonia for the conversion between glutamine and glutamate. However, other mechanisms have been proposed to support ammonia homeostasis in brain, which have not been implemented in the present model and may lessen the requirement for GDH, including diffusion of ammonia as gaseous NH_3_ or the purine nucleotide cycle [71].

An interesting possibility for future research could be the incorporation of the stoichiometry between Na^+^ and K^+^ ionic fluxes and neuronal glutamate release that we report here into larger models of brain metabolism. Indeed, the set of reactions in our model is comparable to some previously published models (e.g., [8]) but much smaller than others (e.g., [9, 12]). Our approach to limit the analysis to the major pathways involved in glutamatergic signaling is equivalent to have assumed that the remaining parts of the metabolic network (not included in the present model) simply adapt to maintain the homeostasis of the metabolic species we considered. Extending large-scale metabolic models of brain metabolism with the information that we have found in the present study may allow to examine the effect of these additional metabolic pathways on the ionic movements underlying neurotransmission, and vice versa.

In conclusion, we have developed a mass-balance model of compartmentalized brain energy metabolism to examine the functional mechanisms underlying the metabolic relationships between neurons and astrocytes. Our analysis allowed the determination of a simple stoichiometric relation between activity-dependent ion and transmitter homeostasis in neurons (63/58 Na^+^ influx/K^+^ efflux per glutamate released). Based on experimental ^13^C-NMR spectroscopy data obtained in rat brain we found that such ratio must be close to 1:1, thereby departing from the the 3/2 Na^+^/K^+^ ratio underlying NKA activity moving Na^+^ and K^+^ in the opposite direction to re-establish ion homeostasis. Thus, stoichiometry alone proved successful in reproducing experimental observations of activity-induced stimulation of astrocytes. These results support the idea [26] that the excess K^+^ mechanism may constitute the basis for astrocytic activation in response to neuronal activity and corresponding neuron-astrocyte functional and metabolic interactions, a phenomenon that should not be neglected in studies aiming at investigating the contribution of these glial cells in brain energetics.

## Electronic supplementary material

Below is the link to the electronic supplementary material.

Supplementary material 1 (PDF 187 KB)

Supplementary material 2 (XML 134 KB)

Supplementary material 3 (PDF 255 KB)

Supplementary material 4 (TIF 1568 KB)

Supplementary material 5 (TIF 19392 KB)

Supplementary material 6 (TIF 2273 KB)

Supplementary material 7 (TIF 2345 KB)

Supplementary material 8 (TIF 2221 KB)

Supplementary material 9 (TIF 2606 KB)

Supplementary material 10 (TIF 2337 KB)

Supplementary material 11 (TIF 2365 KB)

Supplementary material 12 (TIF 2293 KB)

Supplementary material 13 (TIF 2271 KB)

Supplementary material 14 (TIF 2611 KB)

Supplementary material 15 (TIF 2498 KB)

Supplementary material 16 (XLSX 63817 KB)

Supplementary material 17 (TIF 3871 KB)

Supplementary material 18 (TIF 2335 KB)

Supplementary material 19 (TIF 1275 KB)

Supplementary material 20 (TIF 19706 KB)

Supplementary material 21 (DOCX 16 KB)
