# Supplementary material for: Computational Flux Balance Analysis Predicts that Stimulation of Energy Metabolism in Astrocytes and their Metabolic Interactions with Neurons Depend on Uptake of K+ Rather than Glutamate
Source: Neurochem Res. 2016 Sep 14;42(1):202–16. doi: 10.1007/s11064-016-2048-0 (PMC5283516; doi:10.1007/s11064-016-2048-0)
Supplement: Supplementary file 1 — Supplementary material 1 (PDF 187 KB) [file 11064_2016_2048_MOESM1_ESM.pdf]

Electronic Supplementary Material

Computational flux balance analysis predicts that stimulation of energy metabolism in astrocytes and their metabolic interactions with neurons depend on uptake of K^+ rather than glutamate

Mauro DiNuzzo[1,], Federico Giove[2,3], Bruno Maraviglia[2,3], Silvia Mangia[4]*

[1] Center for Basic and Translational Neuroscience, Division of Glial Disease and Therapeutics, Faculty of Health and Medical Sciences, University of Copenhagen, Copenhagen, Denmark

[2] Museo Storico della Fisica e Centro Studi e Ricerche “Enrico Fermi”, Rome, Italy

[3] Fondazione Santa Lucia IRCCS, Rome, Italy

[4] Center for Magnetic Resonance Research, Department of Radiology, University of Minnesota, Minneapolis, MN USA

*** correspondence to**: Mauro DiNuzzo, Ph.D.

Center for Basic and Translational Neuroscience, Division of Glial Disease and Therapeutics

Faculty of Health and Medical Sciences, University of Copenhagen

Blegdamsvej 3B, 24.2.40

2200 Copenhagen N, Denmark

e-mail: mauro.dinuzzo@sund.ku.dk

Online Resource 1

Description of the pathways included in the stoichiometric model

In the following text, we briefly describe the metabolic network used to build our model. Please refer to Table 1 in the main manuscript for details about the stoichiometry of individual reaction/transport processes.

Nutrients supply. Under physiological conditions the brain relies on oxidative metabolism of glucose (reviewed by [1]). (GLC) and oxygen (O_2) are supplied from blood to the brain parenchyma via glucose transporter (GLUT) proteins or simple diffusion, respectively. Diffusion is also responsible for clearance of carbon dioxide (CO_2) produced by energy metabolism. We did not explicitly include cellular constituents of the blood-brain barrier (BBB), such as endothelial cells and pericytes. These cell types are important for BBB formation and maintenance, yet they represent a minor fraction of tissue volume and metabolism. Although lactate (LAC) influx from blood to brain becomes relevant in conditions associated with hyperlactatemia, as does occur during intense exercise, we neglected any LAC transport across the BBB. Similarly, the model does not incorporate influx of ketone bodies, which are used by the brain during conditions associated with hyperketonemia, as happens during fasting or low-carbohydrate diets.

Glycolysis. In neurons and astrocytes, one GLC molecule is processed through glycolysis in the cytosol and converted to two molecules of pyruvate (PYR). Glycolysis also produces two molecules of reduced nicotinamide adenine dinucleotide (NADH) and two molecules of ATP. The first preparatory phase of glycolysis involves two phosphorylation steps, where ATP is initially expended to phosphorylate GLC to glucose 6-phosphate (G6P) by hexokinase (HK) and, after isomerization by phosphoglucoisomerase (PGI), fructose-6-phosphate (F6P) to fructose 1,6-bisphosphate (FBP) by phosphofuctokinase (PFK). One molecule of FBP is reversibly converted by aldolase (ALD) to one molecule of glyceraldehyde 3-phosphate (GAP) plus one molecule of dihydroxyacetone phosphate (DHAP), the latter then isomerized to GAP by triose phosphate isomerase (TPI) to proceed further. The second pay-off phase of glycolysis involves three irreversible steps: the conversion of GAP to 1,3-bisphosphoglycerate (BPG) by glyceraldehyde 3-phosphate dehydrogenase (GAPDH) with concurrent reduction of NAD^+ to NADH, followed by two substrate-level phosphorylations producing two ATP molecules. The first is catalyzed by

phosphoglycerate kinase (PGK), which produces 3-phosphoglycerate (3PG). The second occurs after isomerization of 3PG to 2-phosphoglycerate (2PG) by phosphoglycerate mutase (PGM) as well as 2PG to phosphoenolpyruvate (PEP) by enolase (ENO), and involves conversion of PEP to PYR by pyruvate kinase (PK).

Fates of pyruvate. PYR can proceed to cellular oxidative metabolism in mitochondria. Alternatively, it can be reduced to LAC by lactate dehydrogenase (LDH) with concomitant oxidation of NADH to NAD^+. Eventually, LAC can be shuttled between different cell types via the extracellular space. Both LAC and PYR are carried by monocarboxylate transporter (MCT) proteins.

Tricarboxylic acid cycle. After shuttling through mitochondrial monocarboxylate transporters (mMCT), PYR is converted to acetyl-coenzyme A (ACoA) by pyruvate dehydrogenase (PDH). ACoA enters the tricarboxylic acid (TCA) cycle after condensation with oxaloacetate (OAA) to form citrate (CIT) via citrate synthase (CS). CIT is then isomerized to isocitrate (ISO) by aconitase (ACO). Subsequent reactions within TCA cycle involves two dehydrogenation reactions processing ISO to alpha-ketoglutarate (AKG), which is catalyzed by isocitrate dehydrogenase (IDH), and AKG to succinyl-coenzyme A (SCoA), which is catalyzed by alpha-ketoglutarate dehydrogenase (AKGDH), with concurrent production of NADH or NADPH. SCoA is converted to succinate (SUC) by succinyl coenzyme A synthetase (SCS), and then to fumarate (FUM) by succinate dehydrogenase (SDH) and NADH. FUM is isomerized to malate (MAL) by fumarase. MAL is finally dehydrogenated to OAA by cytosolic/mitochondrial malate dehydrogenase (c/mMDH) to complete the cycle. The three dehydrogenation reactions in the TCA cycle produce three molecules of NADH per ACoA processed. Some of the reactions are catalyzed by either mitochondrial or cytosolic enzymes, whereby carrier proteins translocate specific molecules across mitochondrial membrane. For example, MAL is moved inside or outside mitochondria by the dicarboxylate carrier (DCC), while transmitochondrial transport of CIT and ISO occurs through citrate/isocitrate carrier (CIC), the latter accounting also for the transport of citrate via tricarboxylate carrier (TCC).

Pyruvate recycling. Once processed in the TCA cycle, PYR can be regenerated (recycled) from MAL by cytosolic/mitochondrial malic enzyme (c/mME). This process removes excess TCA cycle intermediates, e.g. after entry of glutamate (see below).

Fatty acid synthesis. We accounted for the first step in fatty acid synthesis (with cytosolic/mitochondrial ACoA shunt), which is represented by the ATP-dependent conversion of cytosolic CIT to ACoA and OAA by ATP citrate lyase (ACL).

Shuttles of reducing equivalents from cytosol to mitochondria. The energy stored in the NADH generated during cytosolic reactions (e.g., glycolysis) is moved into mitochondria in the form of reduced molecules, which occurs through glycerol-3-phosphate shuttle (G3PSH) and/or malate-aspartate shuttle (MAS) systems. MAS is the most efficient system for NADH shuttle in the brain [2] and involves two mitochondrial antiporter proteins, the oxoglutarate carrier (OGC) and the aspartate/glutamate carrier (AGC), plus a transamination reaction and a dehydrogenation reaction (c/mMDH, in common with the TCA cycle) running in opposite directions in cytosol and mitochondria. The transamination by cytosolic/mitochondrial aspartate aminotransferase (c/mAAT), which involves glutamate and AKG, represents one entry point of neurotransmitter glutamate into the TCA cycle. Another mechanism is brought about by interconversion of glutamate and AKG by glutamate dehydrogenase (GDH) occurring in mitochondria. The GDH-catalyzed reaction uncouples glutamate from the MAS, which requires the presence of mitochondrial glutamate carrier (GC) [3].

Mitochondrial respiration. Oxygen-dependent mitochondrial respiration capitalizes the energy stored in NADH molecules to produce ATP within the electron transport chain, which produces the transmitochondrial proton gradient used for ATP synthesis. The accepted stoichiometry for mitochondrial respiration involves five molecules of NADH yielding approximately 15 ATP molecules per O_2 consumed.

Antioxidant system. Mitochondrial respiration generates reactive oxygen species (ROS) that are scavenged by the NADPH-dependent antioxidant system of glutathione (GSH) [4]. In particular, glutathione peroxidase (GPX) detoxifies ROS using monomeric GSH and yielding gluthatione disulfide. The 2 molecules of GSH are regenerated by glutathione reductase (GR) using NADPH as electron donor.

Pentose phosphates pathway. Regeneration of one molecule of NADPH requires processing of one glucose molecule in the pentose phosphates pathway (PPP). Specifically, G6P is channeled into PPP and dehydrogenated twice to yield two molecules of NADPH. G6P eventually re-enters glycolysis as F6P and GAP, with loss of one carbon atom per G6P molecule processed.

Maintenance of membrane potentials. In neurons and astrocytes, ATP is consumed primarily by the ubiquitous NKA. This enzyme accounts for ~80% of total brain energy expenditure, while the remaining is used for housekeeping processes (e.g., protein synthesis) [5-7], which is accounted for by the generic ATPase fluxes. NKA maintains ionic gradients across the plasma membrane, consuming one ATP molecule to move 3 Na^+ ions outside and 2 K^+ ions inside the cells.

Glutamatergic neurotransmission. The excitatory neurotransmitter glutamate is packed into synaptic vesicles by vesicular glutamate transporter (VGLUT). This transport is coupled to vesicular H^+-ATPase, and considering proton leak the corresponding ATP cosumption is estimated to be 1.5 ATP per glutamate [6]. Neuronal electrical activity involves release of glutamate from synaptic vesicles into extracellular space (i.e. neurotransmission) as well as transmembrane Na^+ and K^+ fluxes due to axonal action potentials and synaptic/dendritic potentials. We considered an aggregate of these Na^+ and K^+ fluxes occurring through voltage-gated (presynaptic/postsynaptic) and ligand-gated (synaptic) ion channels.

Transmitter and ion homeostasis. Astrocytes are responsible for clearance of neuroactive compounds from extracellular space by actively taking up glutamate and K^+. Astrocytic uptake of glutamate occurs through excitatory glutamate transporter (EAAT) proteins, which also translocate 3 Na^+ inside and 1 K^+ outside the cell for each glutamate molecule. In astrocytes, glutamate is amidated to glutamine by astrocyte-specific glutamine synthetase (GS) consuming one ATP molecule per glutamate processed. Glutamine is exported to extracellular space by system N transporter (with concomitant exchange of Na^+ and H^+) and taken up into neurons by Na^+-dependent system A transporter. Neuronal glutamine is finally converted back to glutamate by neuron-specific phosphate-activated glutaminase (PAG), within the so-called glutamate-glutamine cycle [8]. The K^+ taken up by astrocytic NKA is returned to neurons through inward rectifying K^+ (KIR) channels [9]. Astrocytes require Na^+ loading to take up K^+, a process that is mediated by voltage-sensitive Na^+ (Nax) channels, among others [10]. We assumed that astrocytic Nax channels also account for net Na+ fluxes mediated by other ion channels, such as Na^+/H^+ exchanger, Na^+/HCO_3^- cotransporter and Na^+/Ca^{2+} exchanger proteins that are not explicitly incorporated in the model.

Ammonia homeostasis. Glutamate-glutamine cycle produces a net transfer of ammonia from astrocytes to neurons. The two major mechanisms for return of ammonia to neurons and ammonia homeostasis occur via trapping of ammonia in alanine (ALA) and/or branched chain amino acids (BCAA) through aminotransferases (either ALAT or BCAT, respectively) plus

intercellular trafficking of these compunds. The ALAT and BCAT enzymes transaminate the amino group from glutamate (forming AKG) to PYR and a branched chain keto acid (BCKA), respectively (forming ALA and a BCAA, respectively) [11, 12].

Anaplerotic reactions of TCA cycle. Anaplerosis mainly takes place in astrocytes. These cells are the only ones capable of replenishment of TCA cycle intermediates directly from glucose (via CO_2 fixation) through pyruvate carboxylase (PC). Requirement of pyruvate carboxylation has been often linked to cerebral glutamine efflux to the bloodstream, but recently it has been showed that, if anything, glutamine is more likely to enter the brain [13]. Anaplerosis could also be the result of citrate release by astrocytes, a mechanism that has been linked to chelation-dependent homeostasis of divalent and trivalent cations (see, for example, [14]). We did not assume any specific pathway for loss of TCA cycle intermediate, as PC activity is indirectly stimulated by oxidative metabolism via NADPH-dependent antioxidant system coupled with pyruvate recyling in mitochondria (see 'Results' section). In particular, the NAPDH required to regenerate GSH is provided by the cytosolic and mitochondrial isoforms of malic enzyme (cME and mME, respectively), which convert malate to pyruvate and CO_2. In turn, pyruvate and CO_2 are used by PC to yield OAA, with no loss of TCA cycle intermediate. The GDH-catalyzed reaction for entry of glutamate into TCA cycle as AKG represents another anaplerotic pathway, which is necessary for ammonia homeostasis (see above).

References

1. Mangia S, Giove F, Tkac I, Logothetis NK, Henry PG, Olman CA, Maraviglia B, Di Salle F, Ugurbil K (2009) Metabolic and hemodynamic events after changes in neuronal activity: current hypotheses, theoretical predictions and in vivo NMR experimental findings. J Cereb Blood Flow Metab 29:441-463
2. Llorente-Folch I, Rueda CB, Amigo I, del Arco A, Saheki T, Pardo B, Satrustegui J (2013) Calcium-regulation of mitochondrial respiration maintains ATP homeostasis and requires ARALAR/AGC1-malate aspartate shuttle in intact cortical neurons. J Neurosci 33:13957-13971, 13971a
3. Mangia S, Giove F, Dinuzzo M (2012) Metabolic pathways and activity-dependent modulation of glutamate concentration in the human brain. Neurochem Res 37:2554-2561

4. Belanger M, Allaman I, Magistretti PJ (2011) Brain energy metabolism: focus on astrocyte-neuron metabolic cooperation. Cell Metab 14:724-738
5. Hyder F, Fulbright RK, Shulman RG, Rothman DL (2013) Glutamatergic function in the resting awake human brain is supported by uniformly high oxidative energy. J Cereb Blood Flow Metab 33:339-347
6. Attwell D, Laughlin SB (2001) An energy budget for signaling in the grey matter of the brain. J Cereb Blood Flow Metab 21:1133-1145
7. Howarth C, Gleeson P, Attwell D (2012) Updated energy budgets for neural computation in the neocortex and cerebellum. J Cereb Blood Flow Metab
8. Chaudhry FA, Schmitz D, Reimer RJ, Larsson P, Gray AT, Nicoll R, Kavanaugh M, Edwards RH (2002) Glutamine uptake by neurons: interaction of protons with system a transporters. J Neurosci 22:62-72
9. Hertz L, Xu J, Song D, Yan E, Gu L, Peng L (2013) Astrocytic and neuronal accumulation of elevated extracellular K(+) with a 2/3 K(+)/Na(+) flux ratio-consequences for energy metabolism, osmolarity and higher brain function. Frontiers in computational neuroscience 7:114
10. Xu J, Song D, Xue Z, Gu L, Hertz L, Peng L (2013) Requirement of Glycogenolysis for Uptake of Increased Extracellular K(+) in Astrocytes: Potential Implications for K (+) Homeostasis and Glycogen Usage in Brain. Neurochem Res 38:472-485
11. Hutson SM, Berkich D, Drown P, Xu B, Aschner M, LaNoue KF (1998) Role of branched-chain aminotransferase isoenzymes and gabapentin in neurotransmitter metabolism. J Neurochem 71:863-874
12. Dadsetan S, Kukolj E, Bak LK, Sorensen M, Ott P, Vilstrup H, Schousboe A, Keiding S, Waagepetersen HS (2013) Brain alanine formation as an ammonia-scavenging pathway during hyperammonemia: effects of glutamine synthetase inhibition in rats and astrocyte-neuron co-cultures. J Cereb Blood Flow Metab 33:1235-1241
13. Bagga P, Behar KL, Mason GF, De Feyter HM, Rothman DL, Patel AB (2014) Characterization of cerebral glutamine uptake from blood in the mouse brain: implications for metabolic modeling of 13C NMR data. J Cereb Blood Flow Metab 34:1666-1672
14. Westergaard N, Sonnewald U, Unsgard G, Peng L, Hertz L, Schousboe A (1994) Uptake, release, and metabolism of citrate in neurons and astrocytes in primary cultures. J Neurochem 62:1727-1733
