# Supplementary material for: Computational Flux Balance Analysis Predicts that Stimulation of Energy Metabolism in Astrocytes and their Metabolic Interactions with Neurons Depend on Uptake of K+ Rather than Glutamate
Source: Neurochem Res. 2016 Sep 14;42(1):202–16. doi: 10.1007/s11064-016-2048-0 (PMC5283516; doi:10.1007/s11064-016-2048-0)
Supplement: Supplementary file 3 — Supplementary material 3 (PDF 255 KB) [file 11064_2016_2048_MOESM3_ESM.pdf]

Electronic Supplementary Material

Computational flux balance analysis predicts that stimulation of energy metabolism in astrocytes and their metabolic interactions with neurons depend on uptake of K^+ rather than glutamate

Mauro DiNuzzo[1,], Federico Giove[2,3], Bruno Maraviglia[2,3], Silvia Mangia[4]*

[1] Center for Basic and Translational Neuroscience, Division of Glial Disease and Therapeutics, Faculty of Health and Medical Sciences, University of Copenhagen, Copenhagen, Denmark

[2] Museo Storico della Fisica e Centro Studi e Ricerche “Enrico Fermi”, Rome, Italy

[3] Fondazione Santa Lucia IRCCS, Rome, Italy

[4] Center for Magnetic Resonance Research, Department of Radiology, University of Minnesota, Minneapolis, MN USA

*** correspondence to**: Mauro DiNuzzo, Ph.D.

Center for Basic and Translational Neuroscience, Division of Glial Disease and Therapeutics

Faculty of Health and Medical Sciences, University of Copenhagen

Blegdamsvej 3B, 24.2.40

2200 Copenhagen N, Denmark

e-mail: mauro.dinuzzo@sund.ku.dk

Online Resource 3

Description of the CLP-based sampling algorithm

Stoichiometric models can be used to simulate medium- to large-scale metabolic networks, as they do not require details (i.e. parameter values) about each reaction or transport process other than the underlying balance between reactants and products. The non-parametric character of stoichiometric models is a great advantage compared with kinetic models, although the applications of stoichiometric models are limited to steady-state analysis [1]. Flux balance analysis (FBA) is a mathematical framework used to calculate solution of the fluxes defined by the reconstruction of a metabolic network. Typically, optimization strategies (e.g. linear programming) represent the underpinning of FBA to cope with the under-determined nature of the problem. Standard FBA requires the minimization or maximization of a mathematical expression called objective function. Biomass production has been the objective function of choice for simulating metabolism of unicellar organisms [2], whereas other choices have been used in simulations of brain energy metabolism, including maximization of ATP production or redox potential [3]. Depending on the context, objective functions might be more or less arbitrary and their definition might require the introduction of parameters and/or assumptions that can considerably affect the results [4]. For these reasons, different methods have been suggested in order to turn FBA from deterministic into probabilistic using, for example, a Bayesian statistical approach [5]. Such approaches tipically introduce a term of uncertainty in mass-balance equations to estimate the posterior probability densities, but still require the introduction of acceptance/rejection criteria (e.g., [6]). Another means to make FBA amenable to statistics is to change the postulates of mass-balance within the so-called Von Neumann’s scheme (for details, see [7] and references therein). To make the sampling of the solutions space feasible, the above-mentioned methods relax or remove the assumption of flux stationarity. The latter can be maintained at the cost of inefficient sampling (e.g., using rejection sampling) or practical limited coverage of the solution space (e.g., using hit-and-run algorithms) [8, 9]. Flux stationarity and efficient sampling are achieved by other sampling methods, such as Artificial Centering Hit-and-Run (ACHR) alogorithm, which however requires to give up the formal guarantee of convergence characterizing Markov chain-based Monte Carlo sampling methods [8, 10].

We developed our metabolic model using constraint logic programming (CLP), which allows determination of the exact domains of fluxes (i.e. the boundaries of the solutions space). We

finally implemented a sampling method (i.e. without definition of objective functions) based on CLP capable, by remaining in the deterministic framework of FBA, of obtaining the distributions of fluxes with both good convergence and high coverage of the solutions space.

A metabolic network is described by the set of mass balance equations:

$$\frac{d\mathbf{c}}{dt} = \mathbf{S} \cdot \mathbf{j}$$

where $\mathbf{c} = c_1,\ldots,c_m$ is the vector of metabolite concentrations, $\mathbf{j} = j_1,\ldots,j_n$ is the vector of metabolic fluxes and $\mathbf{S}_{m\times n}$ is the stoichiometric matrix. The main idea underlying FBA is that the stoichiometry of the metabolic network is the main source of constraints that cells must obey. This entails that the non-equilibrium steady-state of the system can be studied by neglecting the time-dependence of metabolite concentrations, which is equivalent to have:

$$\mathbf{S} \cdot \mathbf{j} = 0$$

The equations underlying FBA can be interpreted as a constraint satisfaction problem (CSP). Indeed, a CSP consists of a finite set of variables (here, metabolic fluxes), a domain of values for each variable (here, real numbers for all variables), and a finite set of constraints involving the variables (here, the metabolic network as well as the boundary conditions). CLP is a branch of declarative programming that provides a powerful framework for constraint reasoning. Specifically, CLP implements solving methods for CSPs in logic programming. These problems involve finding solutions to a number of constraints, whereby each solution is represented by the binding of each variable with a value within the relevant variable domain. Indeed, a constraint problem is under-determined in many cases, which means that the set of constraints has more than one solution. In the case of finite domains, variable binding is based on backtracking search as in classical logic programming [11]. Although different solvers can be more or less efficient, typically the underlying procedure is always complete, being based on systematic search. For uncountable domains (e.g., real numbers), enumerating all solutions is of course not possible. A common approach is to use an optimization criterion that is essentially based on the assumption that one solution is better than another. To this end, a mathematical function involving a combination of variables is minimized or maximized. In standard FBA, a single solution is obtained via linear optimization of an objective function. However, the objective function is arbitrary and system-dependent. Thus, for example, a “reasonable” objective function for brain metabolism is difficult to identify and so is the interpretation of results coming from

objective function optimization. Therefore, in situations involving uncountable domains, but also for very large finite domains, it would be preferable to obtain an estimate for the mass density (i.e. the distribution) of the solutions. In the present work, we used a simple non-systematic, stochastic search strategy for CLP over real numbers (CLP$\Re$) [12], based on iterative sampling [13].

The sampling algorithm is implemented by combining constraint propagation as an instance of forward-checking [14] with random sampling, using the simplest possible scheme (i.e. uniform random) of variable and value ordering [15, 16]. The method can be viewed as a hybridization of pure Monte Carlo methods (see, for example, [17]). However, contrary to Monte Carlo methods, in our approach an individual sample in the *n*-dimensional space is generated in *n* successive steps. The idea is to estimate distributions of fluxes by applying repeated random sampling while benefiting from CLP to reduce the sample space into a sharp enclosure of the region of integration (see below). This feature guarantees that random values always fall inside the relevant variable domains. Specifically, given the sequence of fluxes $\mathbf{j} = j_1,...,j_n$, the algorithm proceeds as follows:

- Determine the initial flux domains $D_1,...,D_n \subseteq \Re$ so that $j_1 \in D_1,...,j_n \in D_n$ by building the initial constraint store from the mass-balance equations of the metabolic network stoichiometry.
- Run an iterative procedure to generate a sample through successive bindings. A sample is an element $s \in D_1 \times ... \times D_n$ that is also a solution if satisfies all given constraints. For each iteration:
    1. Randomly choose a flux j_k (initially $1 \leq k \leq n$) from the set of currently unbound fluxes (with uniform probability).
    2. Bind the chosen flux to a random value s_k within its domain D_k (with uniform probability). This binding is itself a constraint, i.e. a relation among variables that specify the space of possible values of these variables, and as such it is added to the constraint store (constraints are additive).
    3. Propagate the constraint $j_k = s_k \in D_k$ to prune (i.e. update) one or more unbound flux domains and/or bind of one or more unbound fluxes (if the corresponding domains become single-valued). The addition of a constraint

requires that it holds simultaneously with the the conjunction of all existing constraints, otherwise the constraint store becomes inconsistent (i.e. over-constrained). Therefore, the constraint store expands with the new flux binding constraint, a process that reduces flux domains to $D_1^{(k)} \subseteq D_1,...,D_k = \{s_k\},...,D_n^{(k)} \subseteq D_n$.

4. If one or more flux domains are pruned to empty or inconsistent domain (i.e. the algorithm fails to meet current constraints), exit with no solution.

5. If all fluxes are bound (i.e. all domains are reduced to a single value through either binding or constraint propagation), exit and store the solution $s = \{s_1,...,s_n\}$. Otherwise keep the binding of j_k and iterate to perform successive bindings.

- Restart the search (step 1) with all unbound fluxes until the desired sample size is obtained.

See Online Resource 17 for a simple example of the above-mentioned steps underlying the CLP-based sampling strategy.

Forward checking-based algorithms are very popular non-systematic search strategies for solving constraint satisfaction problems, because they allow to detect inevitable failure early. In our case, constraint propagation binds a variable and then removes conflicting values from the domains of all future (unbound) variables. Since the constraint store is expected to change, the CLP$\Re$ system solves the conjunction of arithmetic constraints in the constraint store by performing incremental constraint solving. The knowledge about the domain ($\Re$) as well as the operations on this domain are built into the solver, so that it does not have to rely on generate-and-test procedures. Thus, for example, CLP$\Re$ can deduce that the conjunction of the constraints $j_i > j_j$ and $j_i < j_j$ is false without testing the constraints for any particular value. The solver uses Gauss-Jordan elimination for linear equalities and an adaptation of the Two-Phase Simplex algorithm for linear inequalities, while non-linear constraints are delayed until they become sufficiently linear, e.g. after the binding of some variables [18]. For example, solving linear equality constraints with Gauss-Jordan elimination involves the substitution of parametric variables with equivalent parametric expressions, which eventually allows estimation of non-parametric variables. Inequality solvers can also determine implied equalities from the set of inequalities. CLP$\Re$ uses constraint-handling rules (CHR) [19] in order to rewrite the set of

constraints and obtain simplification of constraints (replacement of constraints with simpler, equivalent ones) and/or constraint propagation (addition of new redundant constraints that eventually results in further simplification). This procedure can also detect a contradictory set of constraints. Overall, constraint simplification and propagation results in the pruning of the domains of one or more variables. Thus, constraint propagation is a mechanism to reduce the space of solutions that will be explored by the searching algorithm.

As an example, consider the following constraint involving 3 fluxes:

$$j_1 + j_2 - j_3 = 0$$

The domain of each flux is pruned using the following contracting operators (e.g., rules) of interval arithmetics:

$$\begin{aligned} D_1 &\leftarrow D_1 \cap D_3 - D_2 \\ D_2 &\leftarrow D_2 \cap D_3 - D_1 \\ D_3 &\leftarrow D_3 \cap D_1 + D_2 \end{aligned}$$

where

$$D_i + D_j = [\inf D_i + \inf D_j, \sup D_i + \sup D_j]$$

and

$$D_i - D_j = [\inf D_i - \sup D_j, \sup D_i - \inf D_j]$$

Importantly, contracting operators are correct (i.e. they do not eliminate solutions), which provides a safe enclosure of the feasible space of solutions.

The present approach to obtain distributions of fluxes for FBA problems is conceptually different from the approaches taken by Heino et al [4] or Martelli et al [7]. The latter are both based on relaxation of steady-state equation to $\mathbf{S} \cdot \mathbf{j} \approx 0$ or $\mathbf{S} \cdot \mathbf{j} > 0$, respectively, i.e. they remove the assumption of strict flux stationarity. Instead, our approach remains in the deterministic framework of FBA. Therefore, in order to obtain an unbiased benchmarking, we compared the performance of CLP-based sampling algorithm with the ACHR sampling algorithm. ACHR is the state-of-the-art algorithm used for flux sampling in FBA problems, and it is implemented in the constraint-based reconstruction and analysis (COBRA) toolbox [20]. The main drawback of these algorithms is that there is no formal proof of convergence of the sampled distribution both

for ACHR (the sequence of iterates is not a Markov Chain) and CLP. Therefore, the performance of these algorithms can only be examined using empirical methods. We perfomed standard convergence analysis (Online Resource 18) as well as empirical convergence diagnostics (Online Resource 19) for CLP and ACHR, the latter run using different parameters (see below).

An important difference between our sampling algorithm and ACHR is that CLP is intrinsically non-parametric. Indeed, CLP does *not* require the specification of (1) “reasonable” priors and starting points to initiate sampling; (2) “large enough” warm-up (i.e. burn-in) periods that are required by the sampler to find the numerically non-zero or non-next-to-zero parts of the distributions; (3) skip parameters (e.g., number of steps per point) that are required by the sampler to to fully explore the solutions space avoiding getting trapped near priors; and (4) tolerance parameters, which are used, for example, to evaluate the distance of the current point from the boundaries of the solution space in order to specify direction of next iterate. These arguments must be taken into account when comparing the sampling generated by the two algorithms. In principle, CLP-based sampling should be compared to ACHR run with no warmup points and no skip. However, in these conditions the convergence of ACHR is highly unsatisfactory (Online Resources 18 and 19). This is a known problem for hit-and-run based algorithms, which may require a very large number of steps to achieve convergence [9]. The CLP-based sampling algorithm exhibits very good convergence, which is achieved by ACHR only when the number of warmup points and steps per point is relatively high (of course, these numbers may depend on the specific problem and cannot be determined a priori). We found that the most critical parameter for ACHR is the number of steps per point, which results in low sample autocorrelation (Online Resource 18 Panel H) and good convergence (Online Resources 19 Panels B,D,F,H). Next, we compared the distributions of fluxes generated by the two algorithms by running ACHR with a high number of both warmup points and steps per point (Online Resources 6-10 for unconstrained model and Online Resources 11-15 for model constrained to awake conditions). In particular, given a sample size of 10,000 points, we set the number of steps per points to 1,000 and that of warmup points equal to sample size (i.e. 10,000). The CLP-based sampling algorithm exhibits much higher coverage of the solutions space than ACHR. This again is a known issue for hit-and-run based sampling algorithms, which do not guarantee a high coverage in a high dimensional system [9]. We tested the ACHR algorithm also by setting the number of steps per point to 10,000 but the results did not change (data not shown). It is noted that the afore-mentioned diagnostics only tests whether a sample distribution converges, but provide otherwise no information on the accurate sampling of the solution space. To examine whether the collection of samples produced by our method can be

safely interpreted as realizations for an underlying distribution, we compared the spread matrices calculated from the solutions produced by CLP and ACHR. Importantly, the two sampling algorithms produces nearly identical spread matrices with only minor quantitative differences (compare Figure 5 and Online Resource 20). Overall, the performances of the non-parametric CLP-based sampling are at least comparable with those of the commonly employed ACHR algorithm, with the advantage of a better coverage of the solution space.

The main limitation of the CLP-based method is that the time necessary to obtain a solution is unpredictable and depends substantially on the specific problem (e.g., number of variables and corresponding domain boundaries). This issue is related to the fact that the forward-checking strategy belongs to the so-called heuristics, which are not guaranteed to succeed [21]. For the present (relatively small) metabolic network, the performance of the algorithm is acceptable, achieving a success rate (i.e. the fraction of iterations producing valid solutions) ranging from 60% to 90% and generating approximately 10 solutions per second on a standard personal computer. This value has to be compared with the computational (CPU) time required by ACHR algorithm to generate a solution, which is 3 orders of magnitude smaller relative to CLP. Nevertheless, as illustrated above, for the present model ACHR requires to skip about 1,000 points for each solution to obtain a reliable sampling of the solution space (although with lower coverage compared to CLP). Once this argument is taken into account, the CPU time of the two algorithms become comparable. It should be noted, however, that the CLP-based method has good potential for improvement. First, since the points generated do not depend on prior iterates (contrary to ACHR), the algorithm can be easily made parallel resulting in performance gain increasing linearly with the number of processors. Second, a substantial optimization can be directed to implement (a) dynamic backtracking mechanisms that, for example, go back to the variable that was responsible for the dead-end instead of restarting, and (b) non-random dynamic variable ordering that, for example, bind first variables with smaller domains or those with more constraints. Third, increases in performance could be obtained by translating the present declarative implementation of the CLP-based sampling algorithm, which was developed in Prolog (an interpreted and relatively slow language) to imperative languages using compiled constraint programming libraries available for e.g., C, Python and Java.

Although the CLP-based algorithm will require further validation in the context of FBA flux sampling, the close agreement in terms of distributions, mean values and correlations of fluxes, between the sampled solutions produced by CLP and ACHR warrants the correctness of the outcomes as well as the interpretation and the main conclusions of the present study.

References

1. Somersalo E, Cheng Y, Calvetti D (2012) The metabolism of neurons and astrocytes through mathematical models. Annals of biomedical engineering 40:2328-2344
2. Feist AM, Palsson BO (2010) The biomass objective function. Current opinion in microbiology 13:344-349
3. Cakir T, Alsan S, Saybasili H, Akin A, Ulgen KO (2007) Reconstruction and flux analysis of coupling between metabolic pathways of astrocytes and neurons: application to cerebral hypoxia. Theoretical biology & medical modelling 4:48
4. Heino J, Calvetti D, Somersalo E (2010) Metabolica: a statistical research tool for analyzing metabolic networks. Computer methods and programs in biomedicine 97:151-167
5. Heino J, Tunyan K, Calvetti D, Somersalo E (2007) Bayesian flux balance analysis applied to a skeletal muscle metabolic model. J Theor Biol 248:91-110
6. Occhipinti R, Somersalo E, Calvetti D (2008) Astrocytes as the glucose shunt for glutamatergic neurons at high activity: an in silico study. J Neurophysiol
7. Martelli C, De Martino A, Marinari E, Marsili M, Perez Castillo I (2009) Identifying essential genes in Escherichia coli from a metabolic optimization principle. Proc Natl Acad Sci U S A 106:2607-2611
8. Megchelenbrink W, Huynen M, Marchiori E (2014) optGpSampler: an improved tool for uniformly sampling the solution-space of genome-scale metabolic networks. PLoS One 9:e86587
9. Schellenberger J, Palsson BO (2009) Use of randomized sampling for analysis of metabolic networks. J Biol Chem 284:5457-5461
10. Kaufman DE, Smith RL (1998) Direction choice for accelerated convergence in hit-and-run sampling. Oper Res 46:84-95
11. Jaffar J, Michaylov S (1987) Methodology and Implementation of a CLP System. In: Lassez JL (ed) Logic Programming Proceedings of the 4th International Conference. MIT Press, Cambridge, MA,
12. Holzbaur C (1995) OFAI CLP(Q,R), Manual, Edition 1.3.3. Technical Report TR-95-09. In. Austrian Research Institute for Artificial Intelligence, Vienna,

13. Langley P (1992) Systematic and nonsystematic search strategies. In: Artificial Intelligence Planning Systems: Proceedings of the First International Conference. Morgan Kaufmann, College Park, MD, pp 145-152
14. Haralick RM, L. EG (1980) Increasing tree search efficiency for constraint satisfaction problems. Artificial Intelligence 14:263-313
15. Nudel B (1983) Consistent-labeling problems and their algorithms: expected-complexities and theory-based heuristics. Artificial Intelligence 21:135-178
16. Gent IP, MacIntyre E, Prosser P, Smith BM, Walsh T (1996) An Empirical Study of Dynamic Variable Ordering Heuristics for the Constraint Satisfaction Problem. In: Freuder EC (ed) Principles and Practice of Constraint Programming. Springer-Verlag, pp 179-193
17. Correia M, Meshcheryakova O, Sousa P, Cruz J (2015) Probabilistic Constraints for Robot Localization. In: Pereira F, Machado P, Costa E, Cardoso A (eds) Progress in Artificial Intelligence. Springer International Publishing, Switzerland, pp 480-486
18. Shostak R (1981) Deciding linear inequalities by computing loop residues. Journal of the Association for Computing Machinery 28:769-779
19. Frühwirth T (1998) Theory and practice of constraint handling rules. The Journal of Logic Programming 37:95-138
20. Schellenberger J, Que R, Fleming RM, Thiele I, Orth JD, Feist AM, Zielinski DC, Bordbar A, Lewis NE, Rahmanian S, Kang J, Hyduke DR, Palsson BO (2011) Quantitative prediction of cellular metabolism with constraint-based models: the COBRA Toolbox v2.0. Nature protocols 6:1290-1307
21. Lee JHM, Leung HF, Stuckey PJ, Tam VWL, Won HW (1996) Using stochastic methods to guide search in CLP: A preliminary report. In: Jaffar J, Yap RC (eds) Concurrency and Parallelism, Programming, Networking, and Security. Springer Berlin Heidelberg, pp 43-52
