# Supplementary figures and images for: Computational Flux Balance Analysis Predicts that Stimulation of Energy Metabolism in Astrocytes and their Metabolic Interactions with Neurons Depend on Uptake of K+ Rather than Glutamate
Source: Neurochem Res. 2016 Sep 14;42(1):202–16. doi: 10.1007/s11064-016-2048-0 (PMC5283516; doi:10.1007/s11064-016-2048-0)

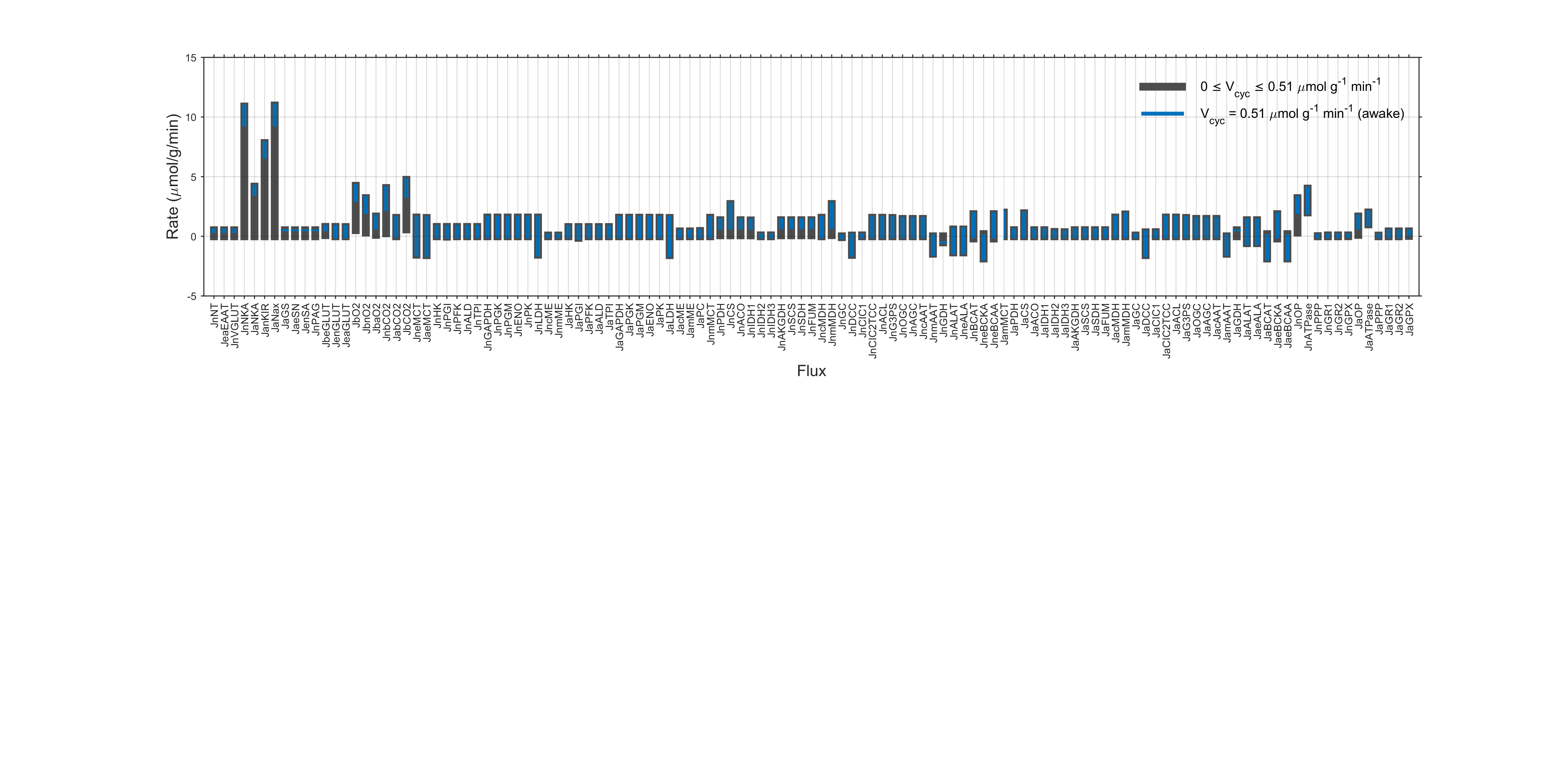

Supplement: Supplementary file 4 — Supplementary material 4 (TIF 1568 KB) [file 11064_2016_2048_MOESM4_ESM.tif]

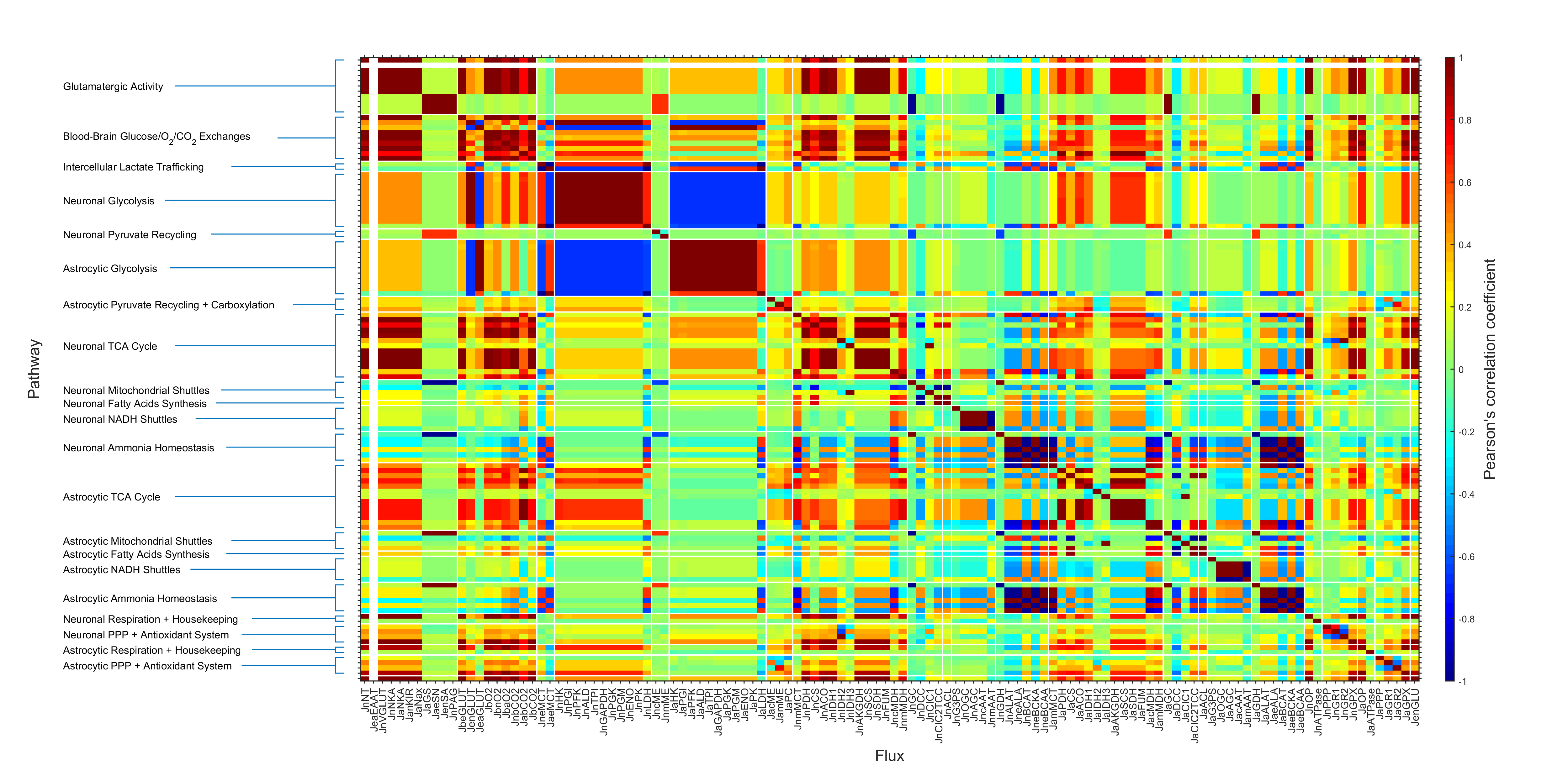

Supplement: Supplementary file 5 — Supplementary material 5 (TIF 19392 KB) [file 11064_2016_2048_MOESM5_ESM.tif]

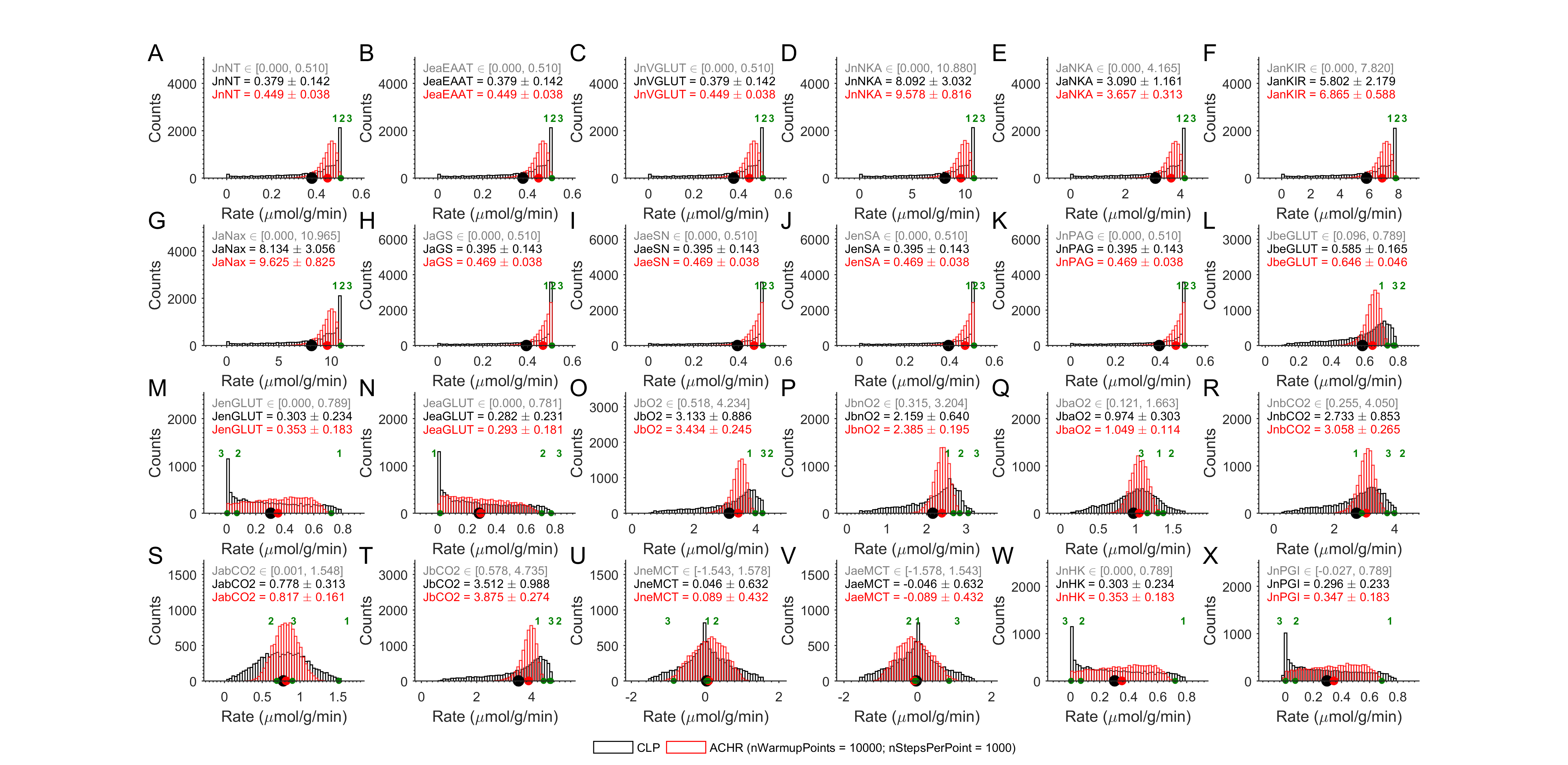

Supplement: Supplementary file 6 — Supplementary material 6 (TIF 2273 KB) [file 11064_2016_2048_MOESM6_ESM.tif]

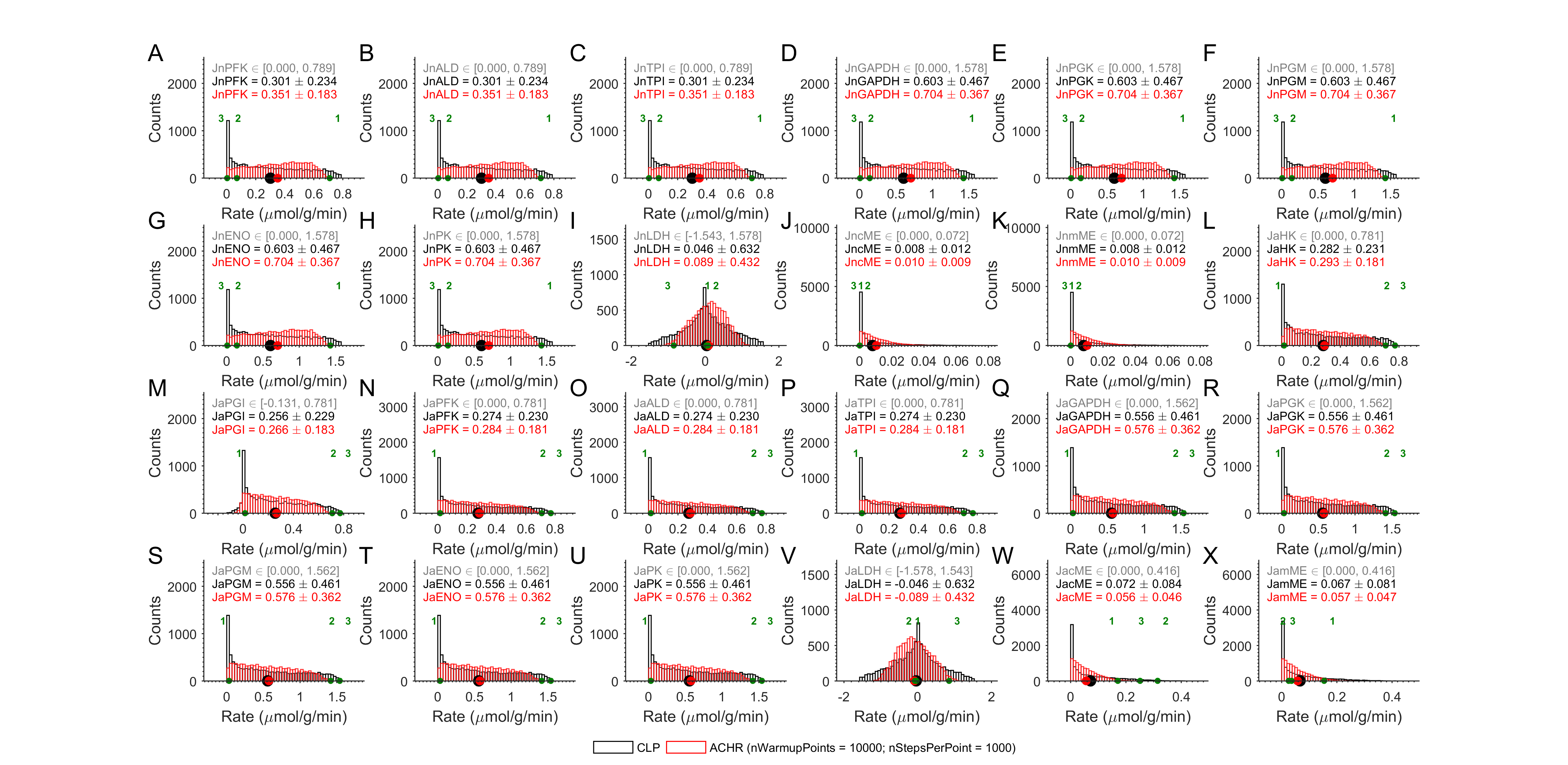

Supplement: Supplementary file 7 — Supplementary material 7 (TIF 2345 KB) [file 11064_2016_2048_MOESM7_ESM.tif]

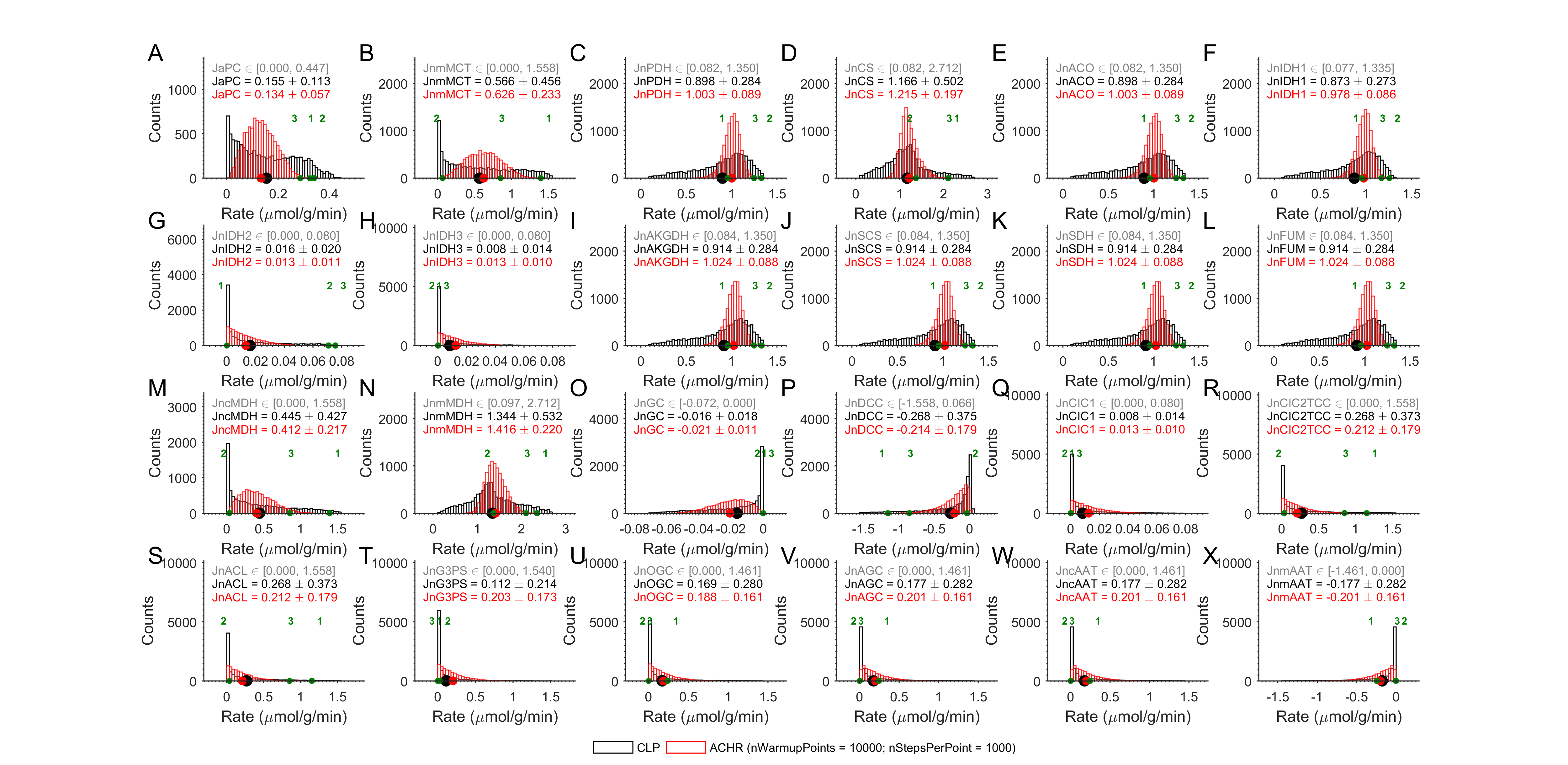

Supplement: Supplementary file 8 — Supplementary material 8 (TIF 2221 KB) [file 11064_2016_2048_MOESM8_ESM.tif]

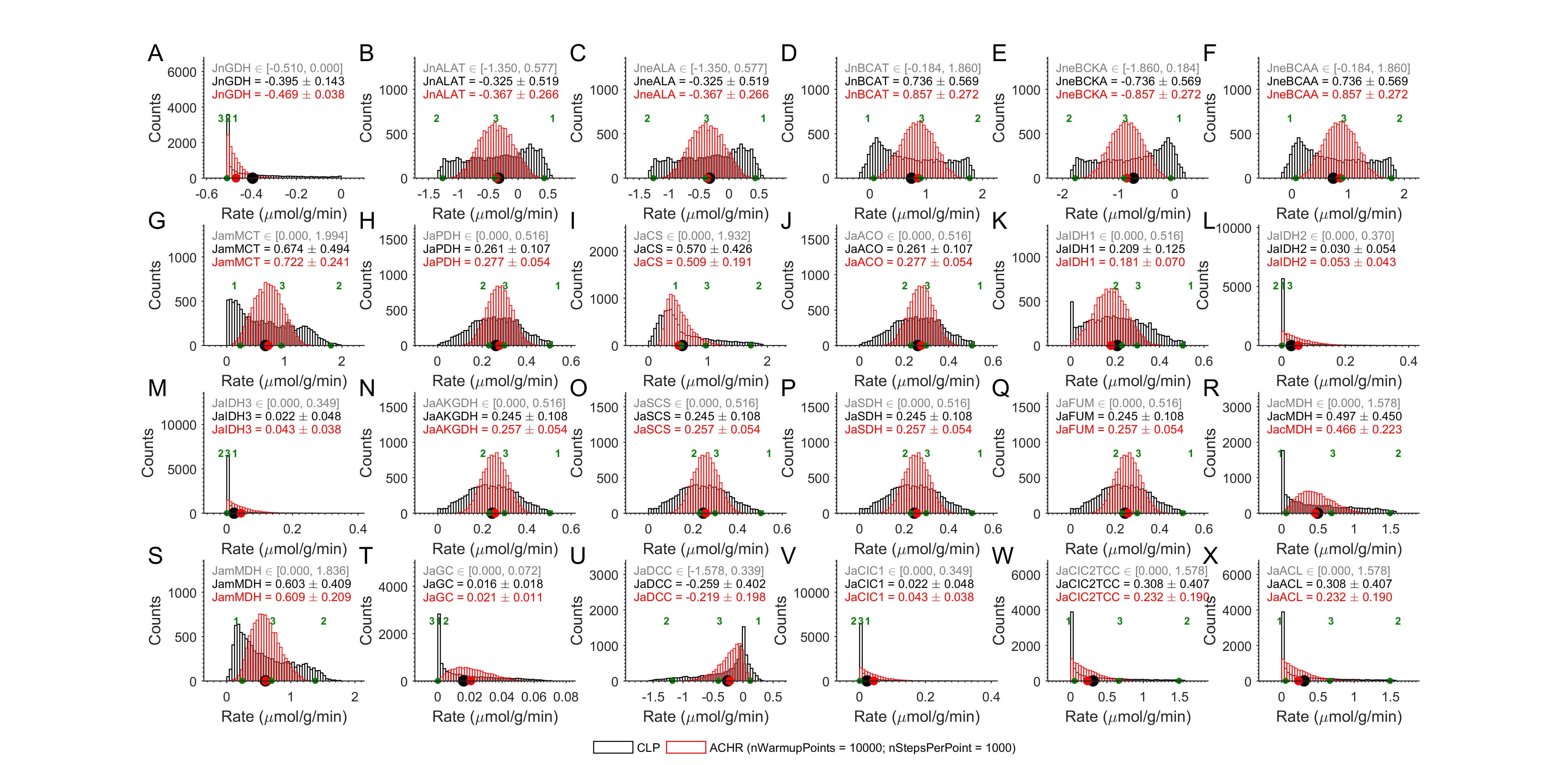

Supplement: Supplementary file 9 — Supplementary material 9 (TIF 2606 KB) [file 11064_2016_2048_MOESM9_ESM.tif]

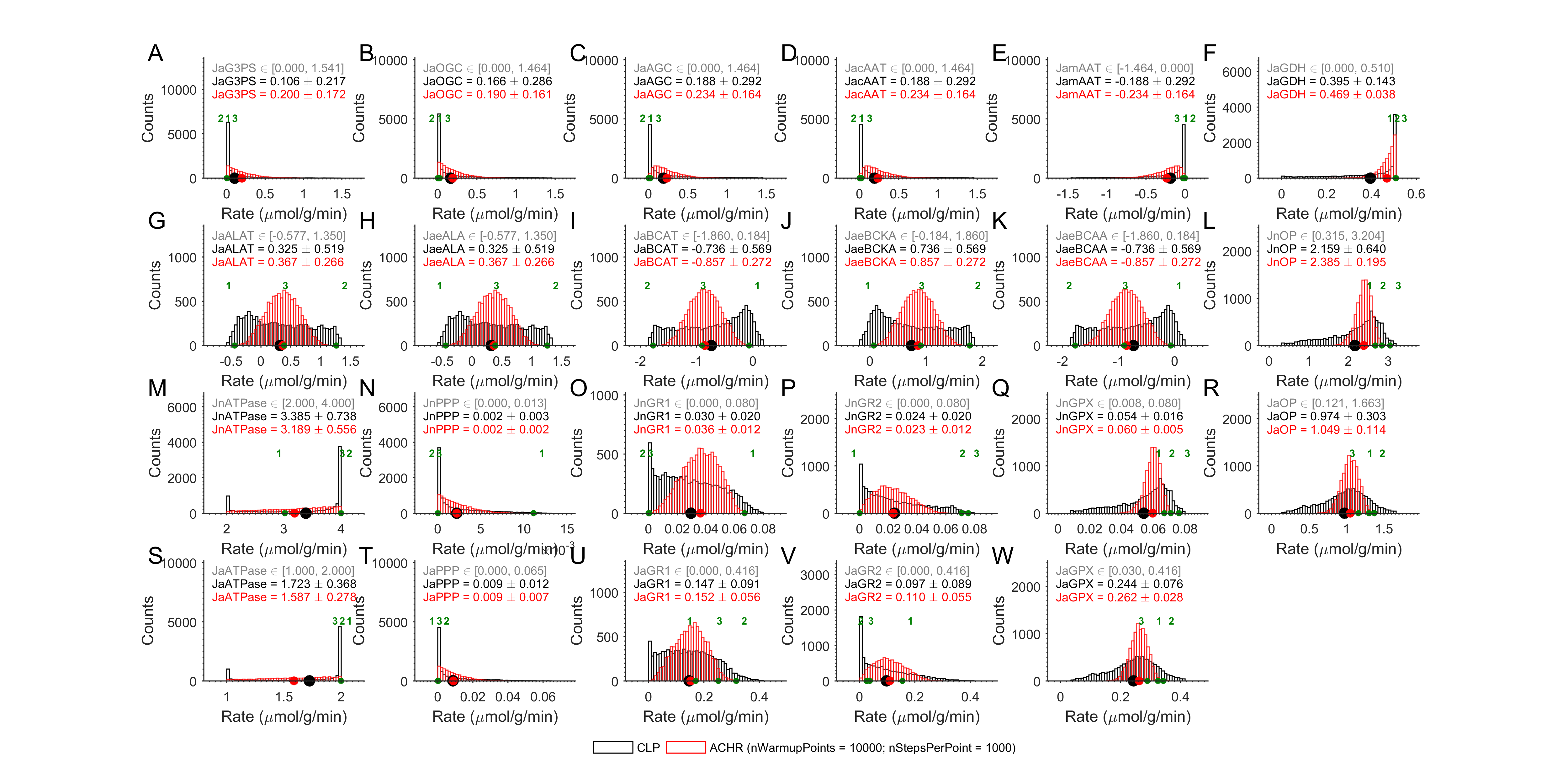

Supplement: Supplementary file 10 — Supplementary material 10 (TIF 2337 KB) [file 11064_2016_2048_MOESM10_ESM.tif]

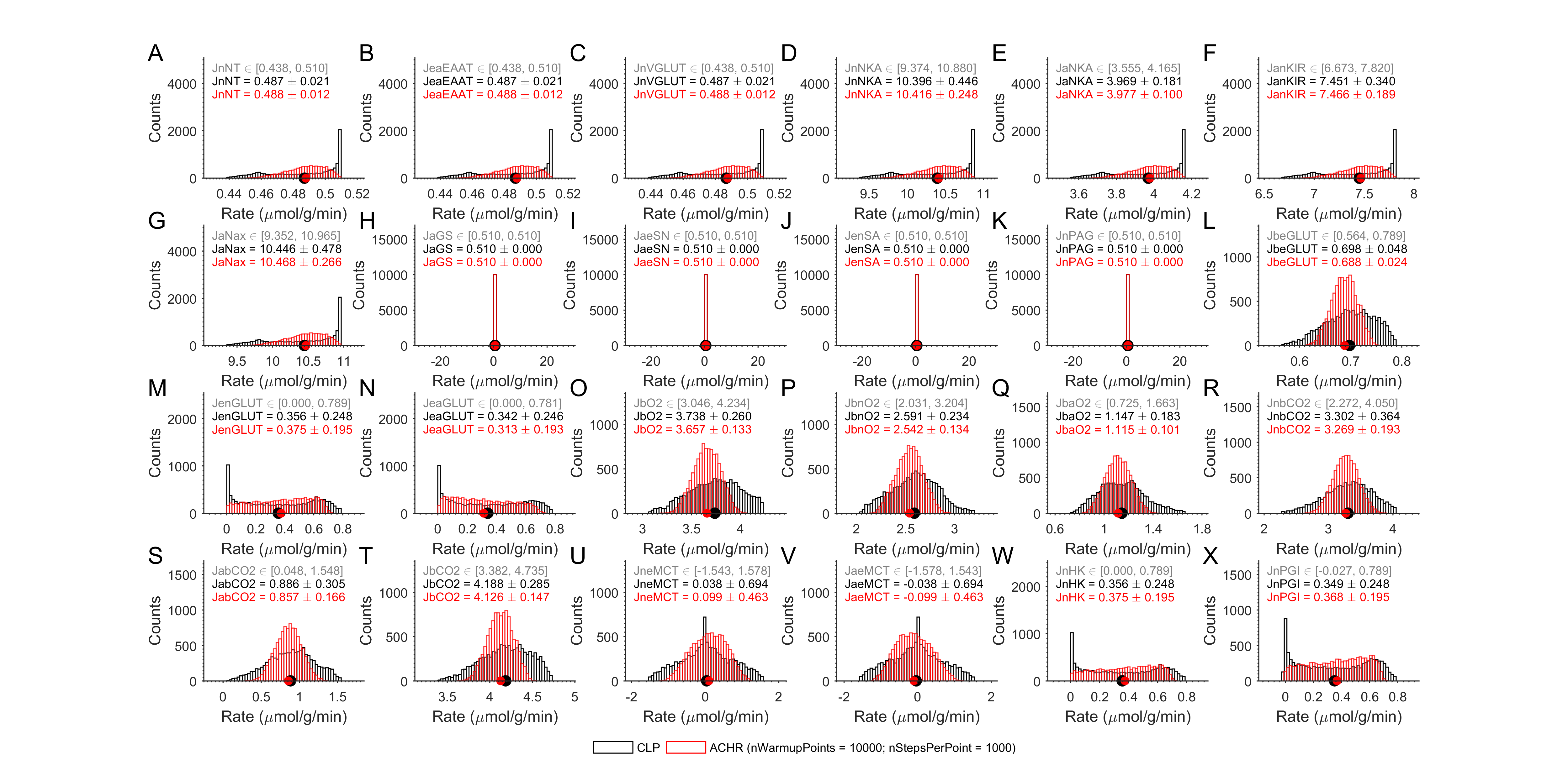

Supplement: Supplementary file 11 — Supplementary material 11 (TIF 2365 KB) [file 11064_2016_2048_MOESM11_ESM.tif]

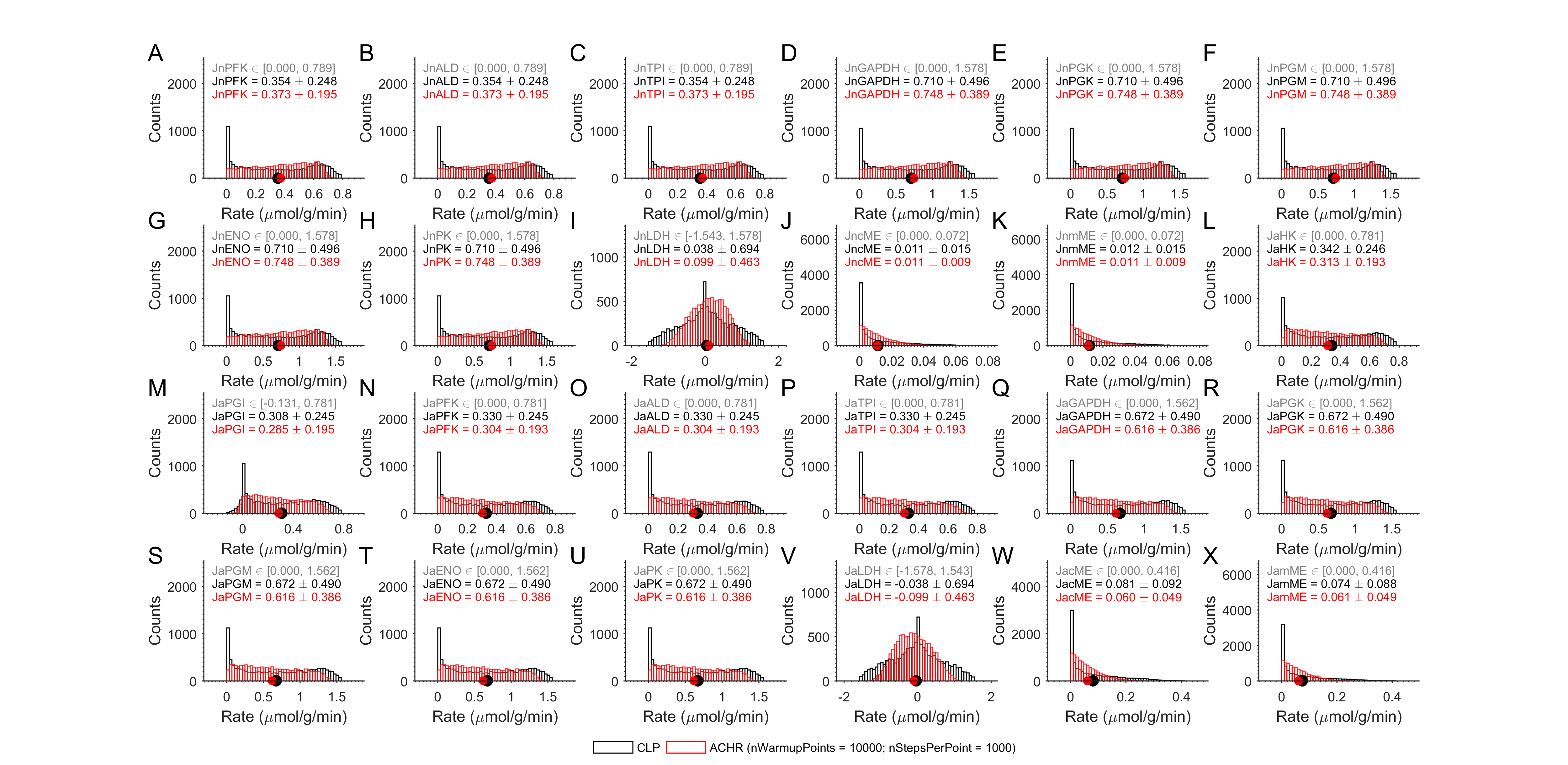

Supplement: Supplementary file 12 — Supplementary material 12 (TIF 2293 KB) [file 11064_2016_2048_MOESM12_ESM.tif]

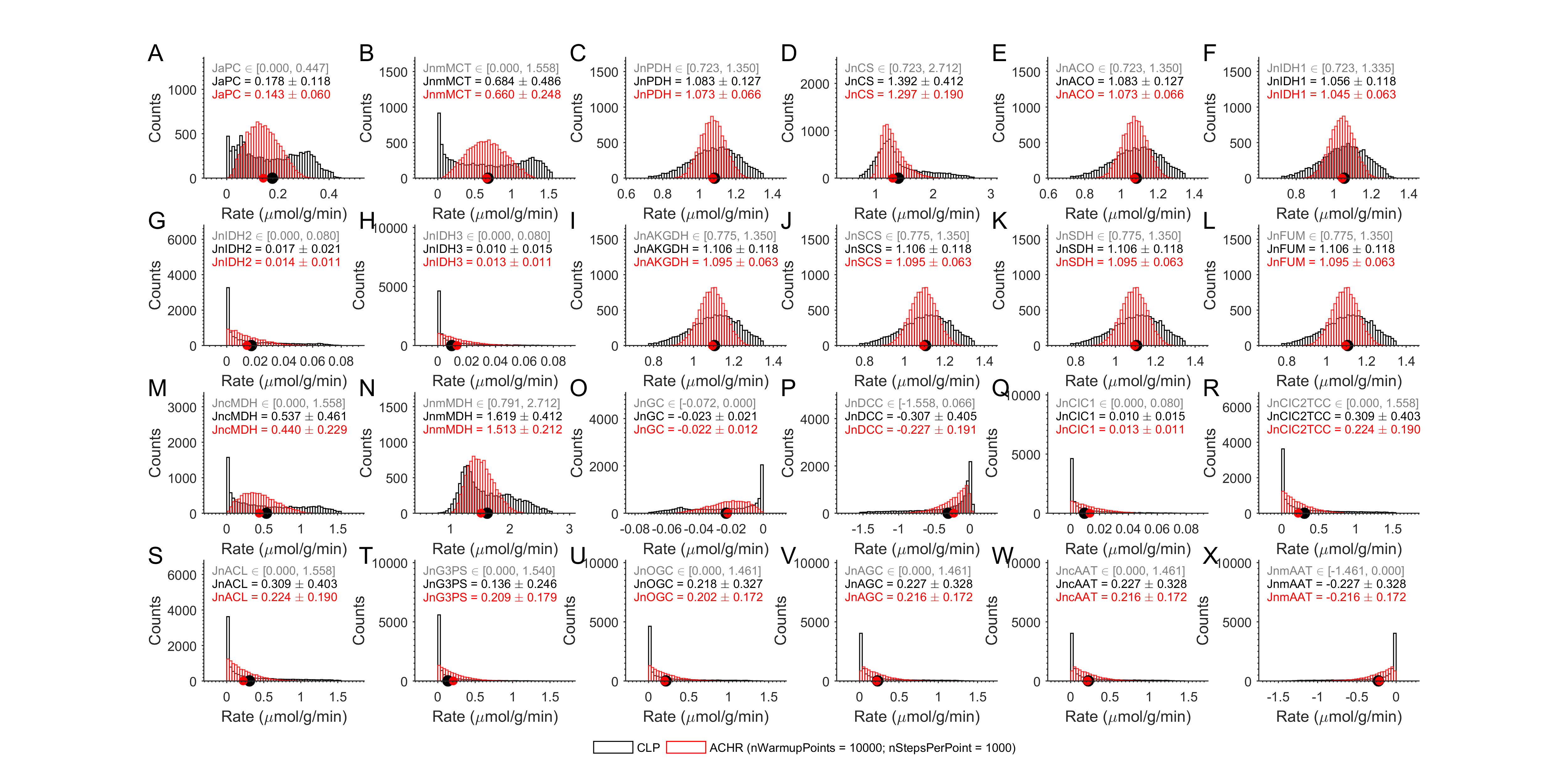

Supplement: Supplementary file 13 — Supplementary material 13 (TIF 2271 KB) [file 11064_2016_2048_MOESM13_ESM.tif]

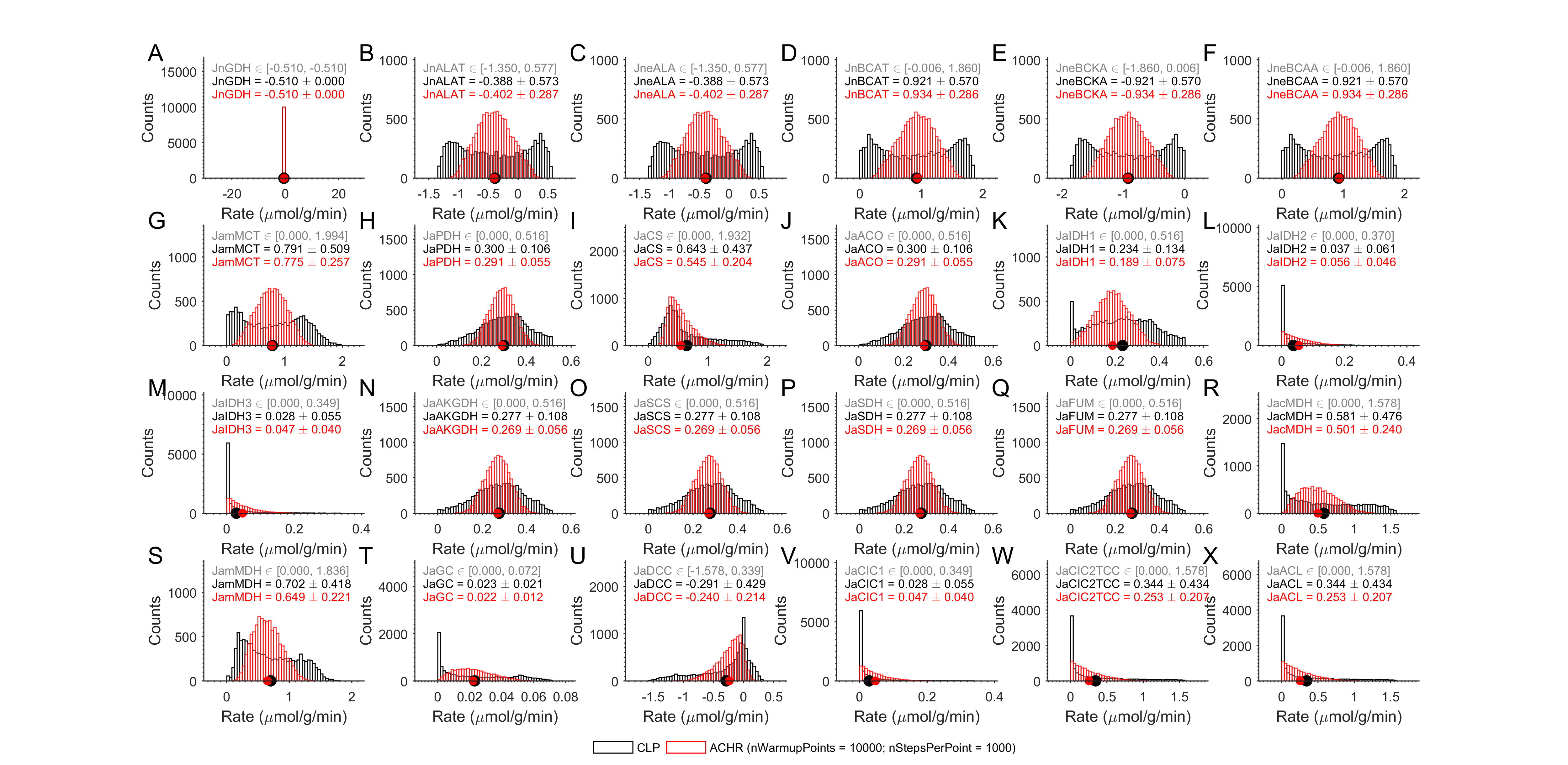

Supplement: Supplementary file 14 — Supplementary material 14 (TIF 2611 KB) [file 11064_2016_2048_MOESM14_ESM.tif]

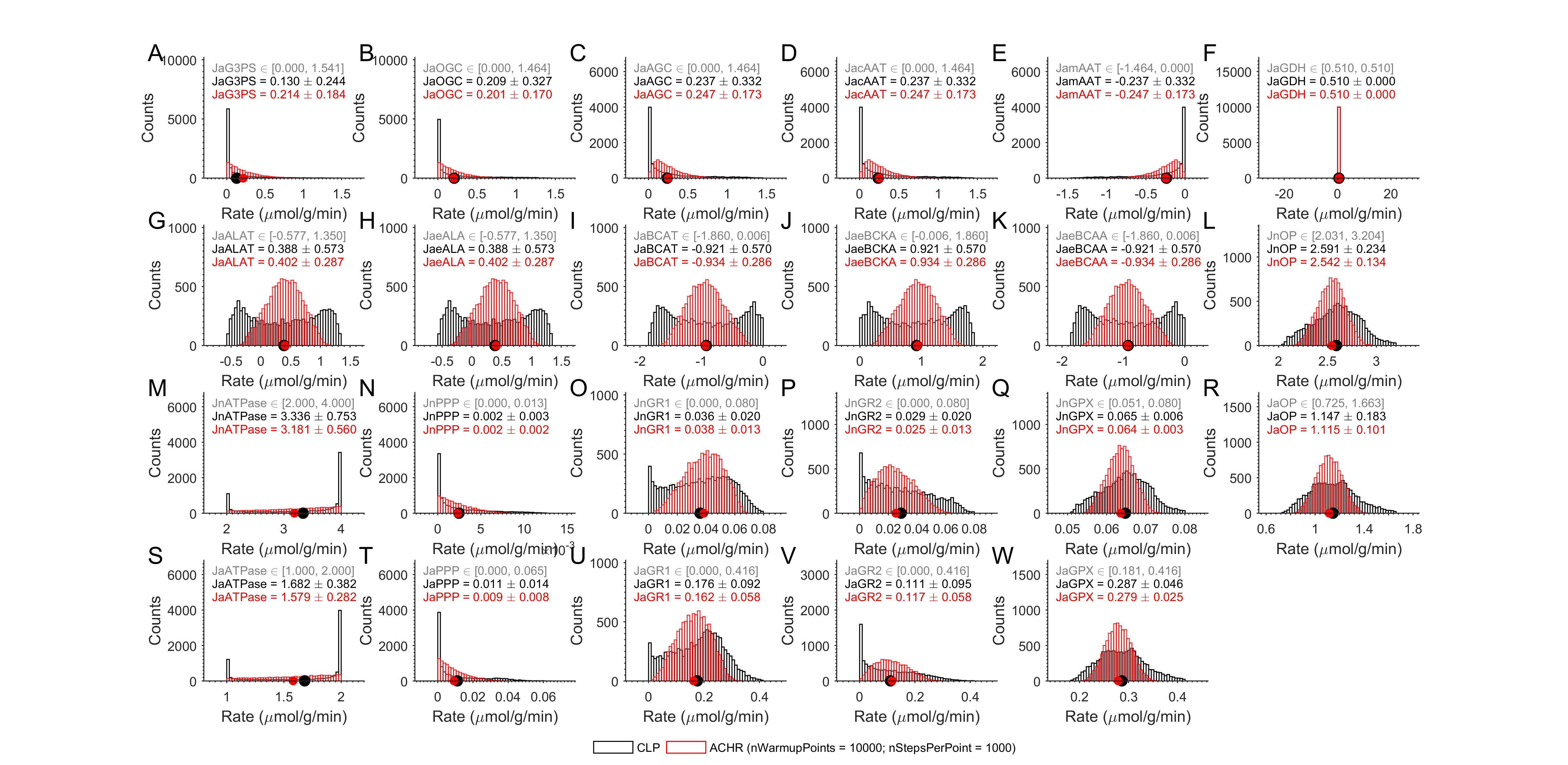

Supplement: Supplementary file 15 — Supplementary material 15 (TIF 2498 KB) [file 11064_2016_2048_MOESM15_ESM.tif]

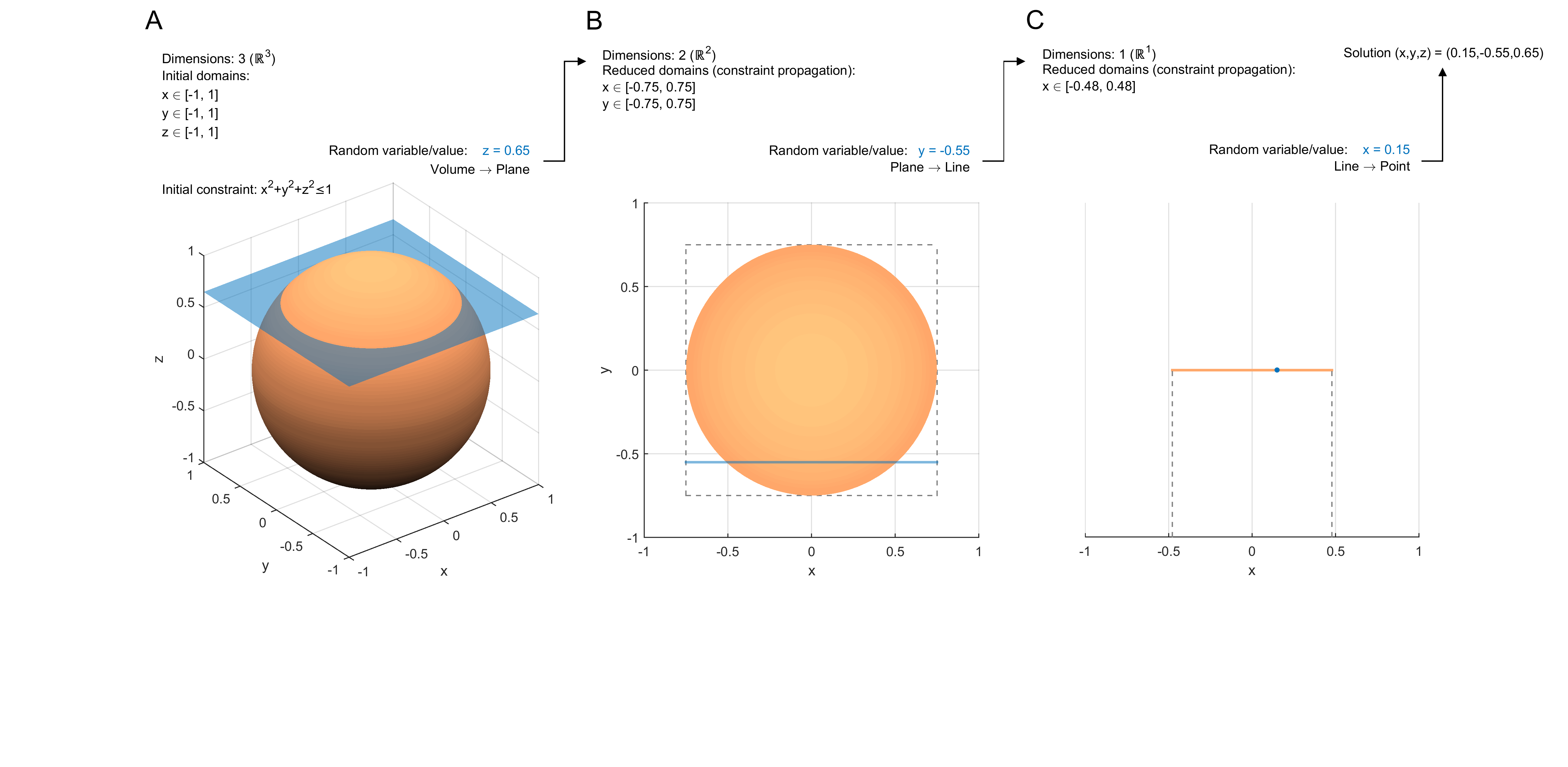

Supplement: Supplementary file 17 — Supplementary material 17 (TIF 3871 KB) [file 11064_2016_2048_MOESM17_ESM.tif]

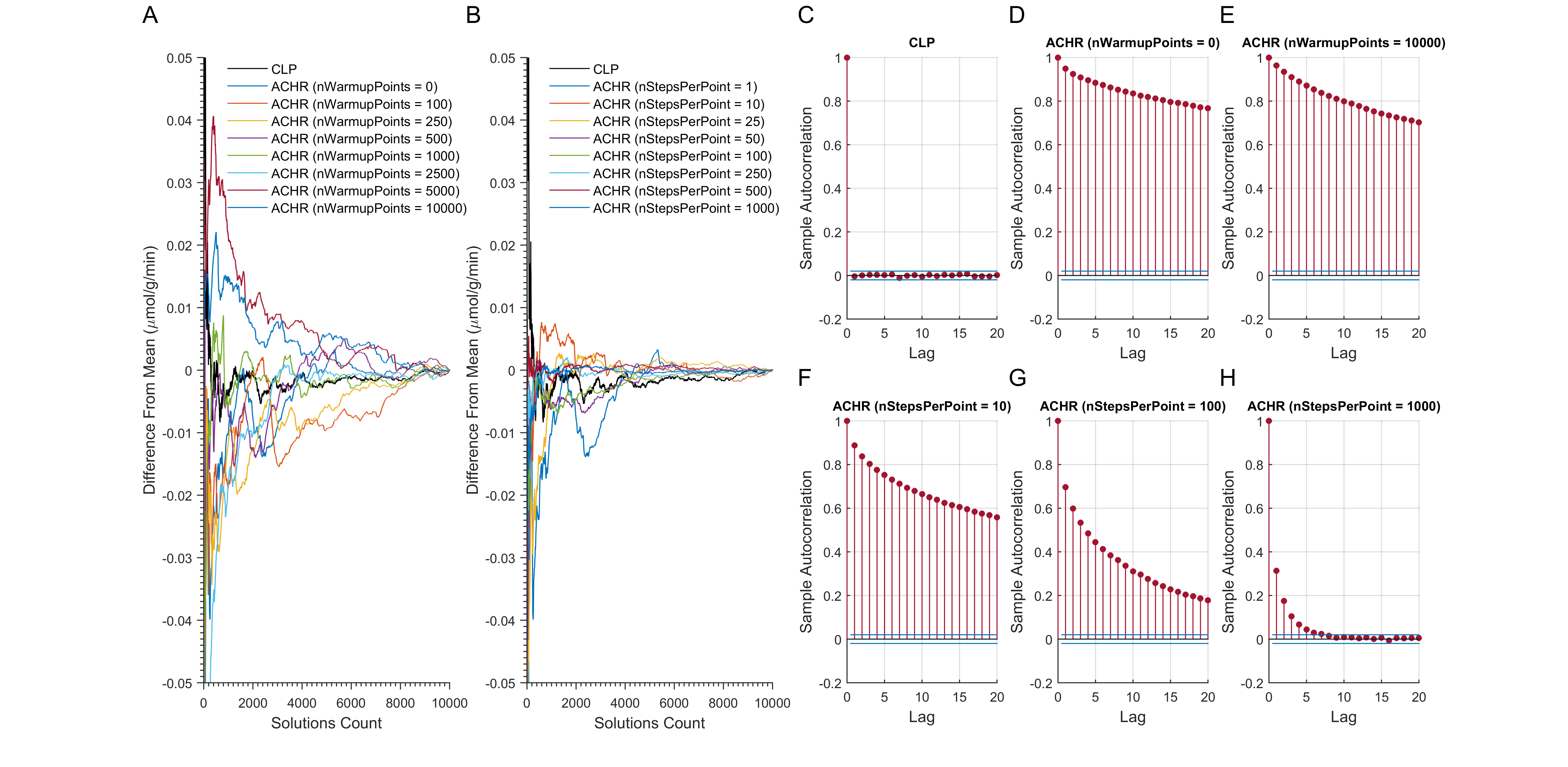

Supplement: Supplementary file 18 — Supplementary material 18 (TIF 2335 KB) [file 11064_2016_2048_MOESM18_ESM.tif]

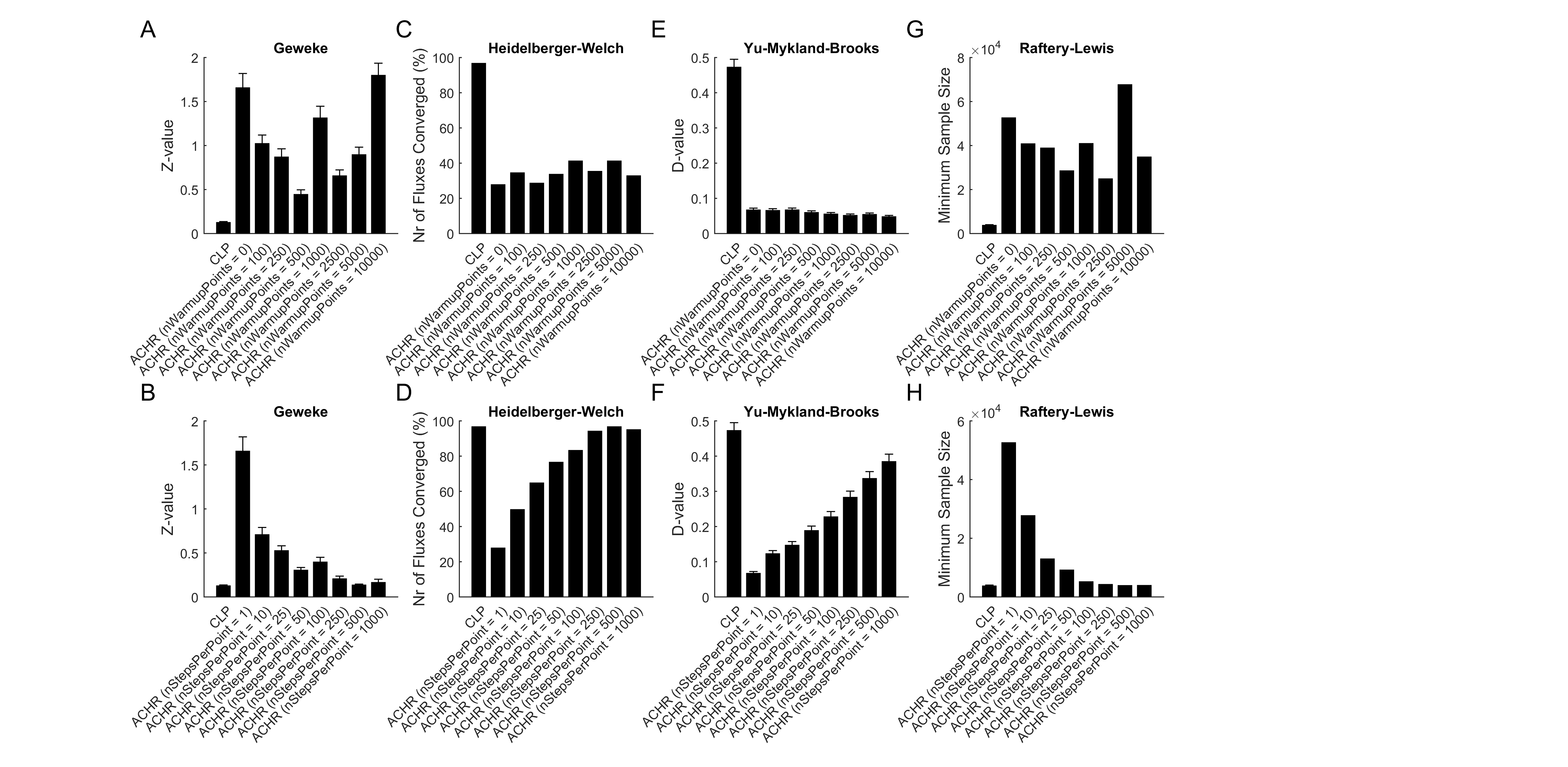

Supplement: Supplementary file 19 — Supplementary material 19 (TIF 1275 KB) [file 11064_2016_2048_MOESM19_ESM.tif]

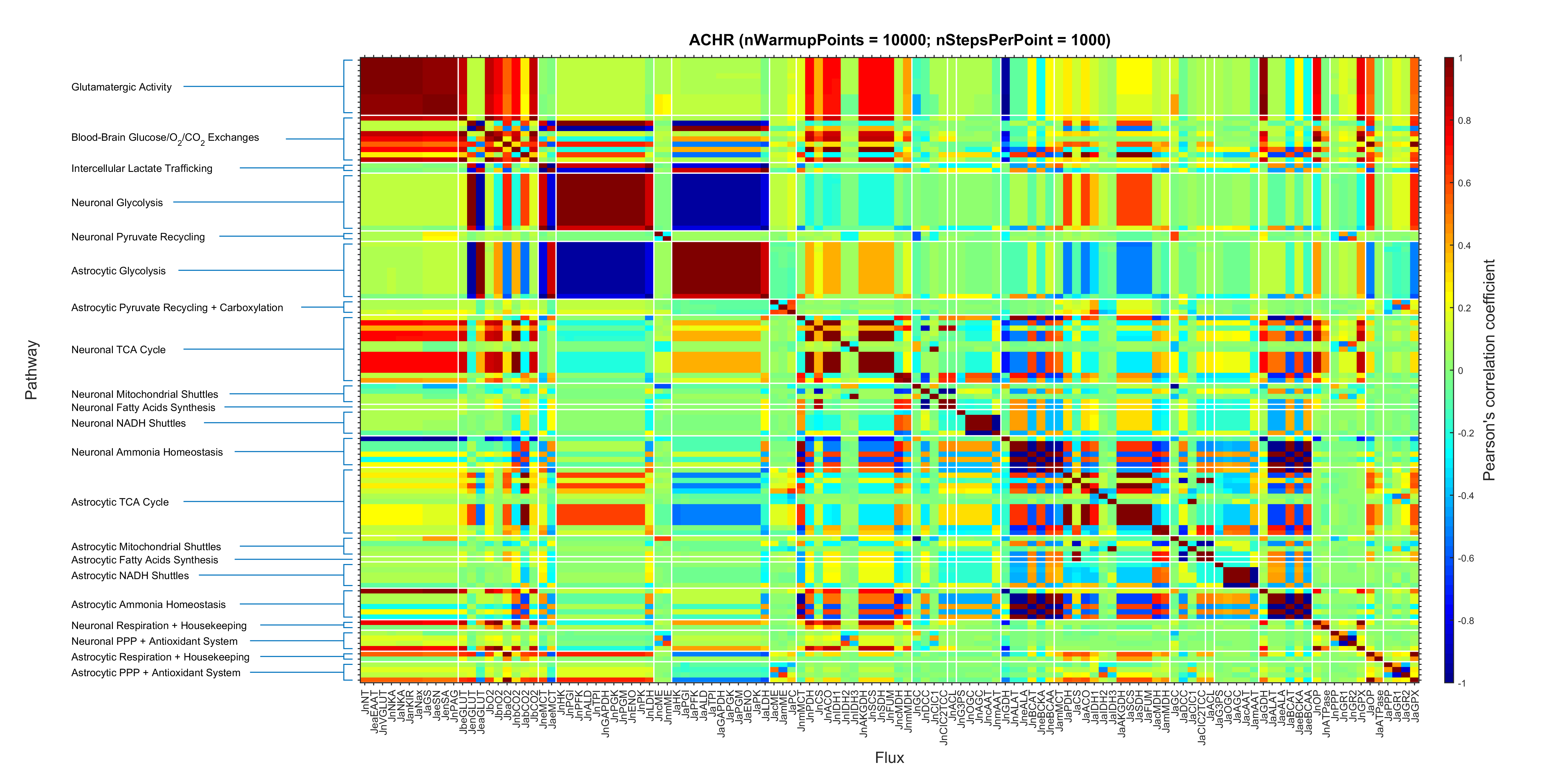

Supplement: Supplementary file 20 — Supplementary material 20 (TIF 19706 KB) [file 11064_2016_2048_MOESM20_ESM.tif]
