# Supplementary material for: Computational Flux Balance Analysis Predicts that Stimulation of Energy Metabolism in Astrocytes and their Metabolic Interactions with Neurons Depend on Uptake of K+ Rather than Glutamate
Source: Neurochem Res. 2016 Sep 14;42(1):202–16. doi: 10.1007/s11064-016-2048-0 (PMC5283516; doi:10.1007/s11064-016-2048-0)
Supplement: Supplementary file 21 — Supplementary material 21 (DOCX 16 KB) [file 11064_2016_2048_MOESM21_ESM.docx]

**Electronic Supplementary Material**

**Online Resource 1.** **Description of the pathways included in the stoichiometric model.** Summary of the reaction/transport processes that form the metabolic network of neuron-astrocyte interactions.

**Online Resource 2. Text file of the reaction/transport fluxes of the stoichiometric model.** The metabolic network model is provided in the Systems Biology Markup Language (SBML) format (level 2, version 4). The file has been used with the COBRA toolbox (http://opencobra.github.io/) for the benchmarking and convergence diagnostic tests (see supporting Text S3). Note that, while reducing the model before sampling, the flag negFluxAllowedFlag (see COBRA documentation) must be set to true.

**Online Resource 3.** **Description of the CLP-based sampling algorithm.** Details about the strategy used to sample the solutions space underlying the FBA problem.

**Online Resource 4. Domains of fluxes compatible with the metabolic network.** Initial constraints (i.e. reaction/transport processes of the metabolic network) prune metabolic flux domains to a large extent (note that initially all variables are real-valued with infinite support), for both the whole sample (black bars) and the sole awake conditions (blue bars). Flux names are reported at the bottom. The actual values are obtained after setting a scaling factor through the constraint 0 ≤ Vcyc ≤ Vcyc^0^. Accordingly, the largest resulting domains (JnNKA and JaNax) correspond to less than 25 times Vcyc, while most fluxes exhibit domains corresponding to 5-10 times Vcyc. This substantial reduction of flux domains allows the use of the constraint propagation iterative technique for the subsequent sampling procedure. Note that positive and negative values indicate forward and backward fluxes. In particular, negative fluxes are possible only for reversible reactions/transports processes.

**Online Resource 5. Spread matrix of model solutions with neuronal but not astrocytic glutamate reuptake.** Without astrocytic EAATs (note the blank row/column in correspondence of JeaEAAT) the correlations between fluxes exhibits a disconnection, within the “Glutamatergic Activity” group, between neurotransmission/ionic currents (JnNT, JnVGLUT, JnNKA, JaNKA, JanKIR, JaNax) and glutamate cycling (JaGS, JaeSN, JenSA, JnPAG), as expected. However, there are only minor changes in the overall correlation patterns of neuronal/astrocytic glycolysis and TCA cycle, suggesting the the neuron-astrocyte metabolic relationships are primarily established because of K^+^ uptake. Note that glutamate reuptake is assumed to be neuronal (see the last row/column in correspondence of the new flux JenGLU not present in the original model). The spread matrix has been determined as in Fig 4.

**Online Resources 6-10. Distributions of metabolic fluxes (unconstrained conditions).** The distributions of fluxes have been computed for unconstrained (0 ≤ Vcyc ≤ Vcyc^0^) conditions by using either the CLP (black histograms) or the ACHR (red histograms) sampling algorithm. The abscissa position of the corresponding circles (at zero ordinate) denote the averages of the relevant distribution, which are reported at the top as mean ± SD. Flux boundaries, as obtained with CLP (Fig S1), are reported at the topmost position in each panel. Remarkably, the two sampling algorithms produce distribution averages that are within the associated errors (in many instances, the averages are nearly indistinguishable). The CLP-based sampling algorithm exhibits a much better coverage of the solutions space compared with ACHR, which is especially apparent near the boundaries of fluxes. All simulated mean flux values are obtained by averaging from a sample of 10,000 model solutions. The green circles correspond to solutions obtained via objective function maximization (1: neurotransmission; 2: ATP production; 3: NADH production). Note that for many fluxes, these optimized solutions fall outside the distributions generated by ACHR but inside the ditributions generated by CLP. Parameters for the ACHR sampling algorithm were nWarmupPoints=10,000 and nStepsPerPoint=1,000. These distributions have been produced using the datasets in Online Resource 16.

**Online Resources 11-15. Distributions of metabolic fluxes (awake conditions).** The distributions of fluxes have been computed for awake (Vcyc = Vcyc^0^) conditions by using either the CLP (black histograms) or the ACHR (red histograms) sampling algorithm. The abscissa position of the corresponding circles (at zero ordinate) denote the averages of the relevant distribution, which are reported at the top as mean ± SD. Flux boundaries, as obtained with CLP (Fig S1), are reported at the topmost position in each panel. The better coverage of solutions space obtained by CLP-based sampling near the boundaries of the distributions is evidenced by the fact that neurotransmission-related fluxes not explicitly constrained (e.g., JnNT and JeaEAAT) give Vcyc^0^ as the most probable value for CLP but not for ACHR. The different coverage of the solutions space of ACHR compared with CLP, together with the close agreement between mean values of fluxes obtained either with CLP and ACHR (see also Figs 7-13) indicates that ACHR remains near the center (i.e. far from both the lower and upper boundaries) of the solution space. All simulated mean flux values are obtained by averaging from a sample of 10,000 model solutions. Parameters for the ACHR sampling algorithm were nWarmupPoints=10,000 and nStepsPerPoint=1,000. These distributions have been produced using the datasets in Online Resource 16.

**Online Resource 16. Archive containing the complete datasets of model solutions (unconstrained and awake conditions).** Data files in the archive (Microsoft Excel spreadsheet format) contain the samples of 10,000 solutions corresponding to simulations performed for unconstrained (0 ≤ Vcyc ≤ Vcyc^0^) as well as for awake (Vcyc = Vcyc^0^) conditions, and obtained either with the CLP or the ACHR sampling algorithm. For all simulations the sum of JnATPase and JaATPase are set to the experimentally determined energy fraction used for housekeeping processes (see ‘Results’ section). Parameters for the ACHR sampling algorithm were nWarmupPoints=10,000 and nStepsPerPoint=1,000.

**Online Resource 17. Schematic representation of the iterative sampling scheme used by the CLP-based algorithm**. Simple example of the iterative steps for reduction of solutions space dimensions, starting from a sphere (A), to a circle (B) and beyond to a line (C). Prior constraint set the initial domains of the variables (A). These domains are progressively pruned during the iterations. In particular, random binding of one variable results in propagation (i.e. forward checking) of the constraint (i.e. the binding itself) and subsequent reduction of the domains of remaining variables (B,C). Finally, random binding of the last unbound variable determines a solution and the cycle starts again. Note that the order of variables for binding is random at each iteration. Note also that the non-linear prior constraint has been chosen only for illustration purposes, as such constraints are not typically present in FBA problems.

**Online Resource 18. Convergence towards mean and sample autocorrelation of sampled points.** The approaching of solutions to their mean values obtained using the CLP-based sampling is compared with ACHR with different settings for the number of warmup points (A) and the number of steps per point (B). In particular, CLP performs better than ACHR for all choices of warmup points (with the minimum skip parameter), while the convergence of the two algorithms are comparable when ACHR is run with sufficiently large step size. Note that the difference from mean was computed for each flux individually and then averaged. In terms of sample autocorrelation, CLP-based sampling (C) behaves better than ACHR (D-H) because two successive solutions produced by CLP are completely independent, which is eventually achieved by ACHR only at very high number of steps per point.

**Online Resource 19. Empirical convergence diagnostics of sampled points.** Different diagnostic tests for examining whether the sampled distribution converges towards a stationary distribution. The CLP-based sampling is compared with ACHR with different settings for the number of warmup points (top panels) and the number of steps per point (bottom panels). The Geweke test (A,B) returns a Z-value for each reaction, with lower values indicating better convergence. The Heidelberger-Welch test (C,D) returns whether the sample distribution for each reaction has converged or not (p<0.005). The Brooks-modified Yu-Mykland test (E,F) returns a D-value for each reaction, with values approaching 1/2 indicating better convergence. The Raftery-Lewis (G,H) test returns, among other values, the minimum sample size required to attain a certain precision (standard ±0.005 degree of accuracy in estimating the 2.5th percentile, p<0.05). The performance of the CLP-based sampling algorithm are very good and comparable to the ACHR when the latter is run with a high number of steps per point. All tests were computed for each flux individually and the relevant results were then averaged.

**Online Resource 20.** **Spread matrix of model solutions produced by ACHR-based sampling.** The correlations between fluxes are in very good agreement with those produced by CLP-based sampling (Figure 4). Specifically, there are no qualitative differences and only minor quantitative differences. Notably, the major pathways involved in glutamatergic signaling, glycolysis and TCA cycle exhibit virtually the same correlations for the two sampling algorithms, both qualitatively and quantitatively. Together with the agreement of distribution average, the consistence between correlation patterns strongly indicates that CLP produces a reliable sampling of the solution space, at least for the present metabolic network. Parameters for the ACHR sampling algorithm were nWarmupPoints=10,000 and nStepsPerPoint=1,000. The spread matrix has been determined as in Figure 4.
